# Guest Binding Mechanism of Polycyclic Aromatic Hydrocarbons by Au(I) Metallo-Tweezers Revealed by Computation

**DOI:** 10.1021/acs.inorgchem.5c03400

**Published:** 2025-11-13

**Authors:** Gantulga Norjmaa, Susana Ibáñez, Eduardo Peris, Jean-Didier Maréchal, Gregori Ujaque

**Affiliations:** † Departament de Química, and Centro de Innovación en Química Avanzada (ORFEO-CINQA), Universitat Autònoma de Barcelona, Cerdanyola Del Vallès, Catalonia 08193, Spain; ‡ Institute of Advanced Materials (INAM), Centro de Innovación En Química Avanzada (ORFEO−CINQA), 16748 Universitat Jaume I, Av. Vicente Sos Baynat S/N, Castellón 12071, Spain

## Abstract

Revealing the mechanisms of supramolecular host–guest binding holds crucial elements for exploring the full potential of supramolecular structures and can lead to further designs and optimizations. Here, we present a computational study of how polycyclic aromatic hydrocarbons (PAHs) bind to a tweezer-shaped molecular receptor (Au­(I) metallo-tweezers) in organic solvents. First, the structure and dynamics of the gold tweezers in solution are characterized with and without the guest molecule bound in the cavity. Second, the guest-binding process is investigated by means of metadynamics simulations. We found that the calculated binding Gibbs energies are in very good agreement with the experimental results, showing the viability of these approaches in the field. Importantly, the study reveals an unanticipated dynamic process that involves spontaneous rotations of the polyaromatic panels of the host, which modulate the size and shape of the cavity until an effective face-to-face arrangement with the planar guest occurs. Once such an interaction occurs, a complete rotation around the carbon-Au bonds finally locates the planar guest molecule in the core of the cavity. This mechanism highlights the variety of dynamic processes that the rich chemical space of supramolecular chemistry can offer.

## Graphical abstract

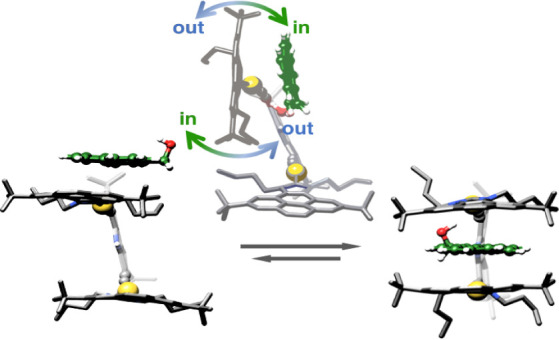

## Introduction

Unveiling the molecular mechanisms behind host–guest interactions is fundamental to advancing the field of supramolecular chemistry.^1–3^ Such understanding requires considering a delicate balance of noncovalent forces—primarily those related to polarity and van der Waals interactions—whose relative contributions vary significantly across systems. While concepts in supramolecular chemistry are often compared to those found in biological systems, the wider chemical space of the former offers interactions and mechanisms with unseen scenarios. This is particularly true when it comes to the role of aromatic interactions. For example, in the 21 amino acid codes of proteins, the strongest components for aromatic interactions are Tryptophan, Phenylalanine, and Tyrosine. These residues offer strong directional hydrophobic interactions (i.e., π-stacking, edge-to-face, etc.) that can dramatically influence the structure and dynamics of guest binding. A prototypical example is tryptophan clamps, which are known to trap planar aromatic moieties.^4–6^

In recent decades, numerous supramolecular architectures have been developed.^6–9^ Depending on their shapes, these structures are often referred to as capsules, cavitands, cages, barrels, tweezers, and so on.^10–13^ Some of these constructs feature aromatic elements that resemble biological motifs, albeit with diverse differences. A notable example is the so-called molecular tweezers, which consist of U-shaped molecular receptors with an open structure, featuring two flat, typically aromatic, identical arms that are connected by a tether, forming a cavity between them.^13–16^ Over the past 20 years, molecular tweezers with metal centers have gained significant attention, not only for their simpler modular synthesis compared to organic counterparts but also for their enhanced photophysical and electrochemical properties, which expand their potential for a broader range of practical applications.^17–37^ One interesting example is the gold­(I)-based metallo-tweezers designed and synthesized by some of us, which feature two pyrene-imidazolylidene-Au­(I) fragments connected by rigid bis-alkynyl spacers. During our research, we discovered that the recognition properties of these metallo-tweezers are strongly influenced by the nature of both the rigid spacer and the ancillary ligands that constitute the arms of the tweezer.^38–44^ The combination of both the spacer and arms establishes a delicate balance between the self-aggregation tendencies of the tweezers and their ability to effectively encapsulate planar guests. In particular, the carbazolyl-bridged metallo-tweezer shown in Figure 1 is capable of effectively recognizing polycyclic aromatic hydrocarbons (PAHs).^44^ Because the shape of this molecule suggests a flapping motion of the polyaromatic hands to facilitate substrate recognition, we considered that a detailed study of this dynamic process would facilitate an understanding of the recognition abilities of this receptor.

**Figure 1:**
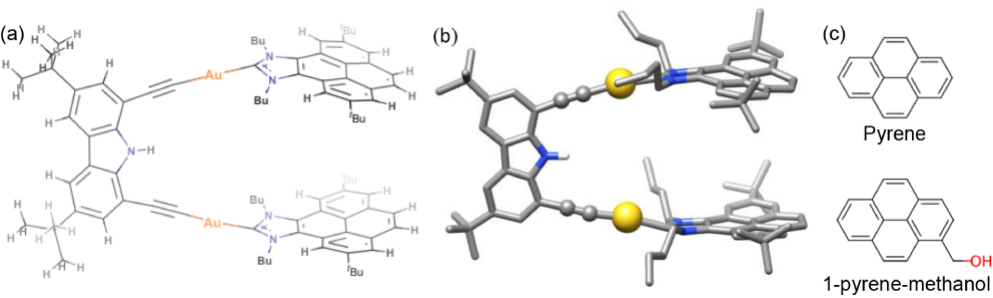
Schematic (a) and molecular (b) representation of bis­(Au-NHC)-based metallo-tweezers. (c) Guest molecules studied in the present work.

Experimentally, this metallo-tweezer exhibits significantly higher binding affinities for planar aromatic guests that are functionalized with hydrogen-accepting groups compared to guests lacking these functionalities. We herein investigate the binding mechanism of both unfunctionalized and functionalized polycyclic aromatic hydrocarbons (PAHs) to Au-tweezers in solution, using two solvents: methanol and toluene. In the present study, a combination of quantum mechanics (QM), classical molecular dynamics (MD), and metadynamics (metaD) simulations^45–47^ were employed to gain insight into the recognition mechanism employed by these molecular tweezers. As will be described below, this analysis provides a detailed picture of the binding process and how these tweezers selectively interact with their guests.

## Computational Details

### Density Functional Theory Calculations

DFT geometry optimizations of the host (Au-tweezers) and the guest (pyrene and 1-methanol pyrene) were performed at the B3LYP-D3 level with the SMD continuum solvent model (toluene as the solvent) using the Gaussian 09 program.^48–53^ The SDD pseudopotential, along with a set of f polarization functions, was used for gold, and the 6-31G­(d) basis set was used for the main group elements.^54^ For the comparison of the Gibbs energy between different binding modes of the host–guest complex, single-point calculations were performed with the 6-311+G­(2d,2p) basis set based on the optimized geometries to obtain more accurate energies. This level of theory (B3LYP-D3/SDD) has been shown to properly describe related gold systems in supramolecular environments.^55^ Gibbs energy corrections were evaluated at the same level of theory as the geometry optimizations using the quasi-rigid-rotor-harmonic-oscillator (quasi-RRHO) approach at 298.15 K with a cutoff of 100 cm^–1^ using the GoodVibes program.^56,57^

### Derivation of Nonstandard Parameters

The metallo-tweezers contain two Au­(I) centers. The parameters for classical molecular dynamics (MD) simulations for these two centers were derived based on Seminario’s method using the Python-based Metal Center Parameter Builder (MCPB.py) program.^58^ The parameters of the bonding terms for the organic moieties of the metallo-tweezers and for the guests were taken from the General AMBER Force Field (GAFF).^59^ van der Waals parameters were obtained from the optimized potentials for liquid simulations force field (OPLS), except for those of Au­(I) taken from the universal force field (UFF).^60,61^ Atomic charges were obtained by fitting the molecular electrostatic potential computed at the quantum mechanics (QM) level according to the restrained electrostatic potential (RESP) method.^62^ To this end, we optimized the metallo-tweezers at the density functional theory (DFT) level, as described above. The AM1-BCC charges were used for the guests, and the Antechamber program was used to assign atom types and derive atomic charges.^100,63^

### Molecular Dynamics Simulations

Classical MD simulations were performed using the CUDA version of the pmemd program from the AMBER 16 package.^64^ For the MD simulation of the metallo-tweezers in the absence of the guest, a simulation box with a size of 65 × 65 × 65 Å, containing metallo-tweezers and ∼3200 methanol or ∼1500 toluene molecules, was treated under periodic boundary conditions. The simulations were performed at a constant temperature (298.15 K, using a Langevin thermostat) and pressure (1 bar, using a Monte Carlo barostat).^65^ The long-range electrostatic interactions were accounted for using the PME method, and a cutoff of 9 Å was applied for nonbonded interactions.^66^ A time step of 2 fs was used in the plain MD simulations. Prior to the production run of 200 ns for each simulation, NPT equilibration of 10 ns was performed after 100000 minimization cycles and a short equilibration of 60 ps. The most populated structures for each simulation were obtained by clustering 5000 MD snapshots using the UCSF Chimera program.^67^ The NMRCLUST algorithm was employed.^68^ The clustering was performed with the default settings (excluding solvents and hydrogens), except the step size was set to 1 to include all MD snapshots.

### Metadynamics Simulations

The well-tempered metadynamics (WT-MetaD)^69^ simulations with multiple walkers^70^ were conducted using the GROMACS 2021.4 software patched with PLUMED 2.8.0.^71^ The AnteChamber Python Parser interfacE (ACPYPE) tool was used to convert the AMBER topologies to GROMACS format. Before the production run, NPT equilibration of 20 ns was performed after a maximum of 2000000 minimization steps. The WT-MetaD simulations were performed along a collective variable (CV), defined as the distance between the center of mass (COM) of the guest and the N atom in the center of the host (see Figure 4). For analytical purposes, WT-MetaD simulations were also performed using a CV defined as the distance between the COM of the guest and the COM of four selected C atoms (two carbon atoms for each pyrene fragment of the host) located on the tweezers (see Figure S1). In these simulations, the height of the deposited Gaussians was 0.5 kJ/mol, and sigma was set to 0.04 nm. Bias was deposited every 5000 steps (10 ps of simulation time). The WT-MetaD simulation with 6 walkers over 100 ns was performed, and the sum_hills function was used to obtain the potential of mean force (PMF). The bias factor was set to 10. The evolution of the collective variable, Gaussian hills deposited during the simulation, and the block analysis to estimate uncertainties are shown in the Supporting Information (Figures S2–S7). The potential of mean force *w*(r) was employed to obtain the binding free energy by integrating over the profile according to

$$\Delta G=-RT\;\ln\left[\int_{0}^{c}4\pi r^{2}e^{-\beta w\left(r\right)}dr\right]$$

where *β* = (*RT*)^−1^ and “*c*” is the cutoff limit that defines the association.^45,72^

*NCI analysis.* The NCI analysis provides an index (based on the electron density and its derivatives) that enables the identification of noncovalent interactions.^73^ The NCI index is based on a 2D plot of the reduced density gradient, *s*, and the electron density, *ρ*. The reduced density gradient is given by

$$s=\frac{1}{2{\left(3\pi^{2}\right)}^{1/3}}\cdot \frac{\left|\nabla \rho \right|}{\rho^{4/3}}$$

where *ρ* is the electron density.^73^

The method bases its estimations on computing the reduced gradient of the electron density (s) versus the electron density (ρ) multiplied by the sign of the second Hessian eigenvalue (λ2), i.e., ρ × sign­(λ2). The Non-Covalent Interaction Analysis (NCI) reported in this work was carried out with the program NCIPlot.^74^ NCIs were first computed using the DFT electron densities, and for clarity, the representation of the isosurfaces was constructed at the molecular level, reporting the density map.

## Results and Discussion

In the following sections, we will first examine the structure and dynamics of the metallo-tweezers in two solvents: methanol (used for their synthesis) and toluene (in which binding affinities were measured). Then, we will discuss and compare the results obtained from molecular dynamics (MD) simulations of host–guest complexes. Finally, we will present the results of the metadynamics simulations in order to evaluate the energetics of the binding process and offer insights into the experimental findings.

### Characterization of Metallo-Tweezers in Solution

The metallo-tweezers were experimentally obtained by deprotonating di*tert*-butyl-diethynyl-carbazole with NaOH in methanol, followed by the addition of the pyrene-imidazolylidene-gold­(I) complex.^44^ We started by analyzing the structure of the metallo-tweezers in explicit solvents (methanol and toluene) using classical MD simulations without a ligand. The simulations show a very broad conformational space, with the pyrene moieties rotating mostly freely around the C–Au bond.

It is interesting to analyze the movement of each pyrene unit of the metallo-tweezers. To do so, we selected two carbon atoms on each pyrene unit: C1 and C2, C3 and C4 (which are the ones adjacent to the *tert*-butyl fragments), and C5 and C6 for the carbazole spacer. These atoms define the dihedral angles between each pyrene fragment (plane A or plane B) and the carbazole connector, which were analyzed during the simulations (Figure 2a). Interestingly, the analysis showed that planes A and B are capable of rotating completely around the C–Au bond in both solvents (Figure 2b,c). This result clearly illustrates that this metallo-tweezer shows a very flexible dynamic behavior. We also calculated the potential energy surface (PES) at the DFT level for the rotation of the host arm using a reduced model, showing an energy barrier of approximately 0.1 kcal/mol (see the Supporting Information, Figure S8). We then performed a cluster analysis of the generated structures along the MD, but as expected, no clear conformational preference was observed. It is worth noting that the most populated structure (less than 2%) resembles that obtained from the experimental single-crystal X-ray analysis.^44^

**Figure 2:**
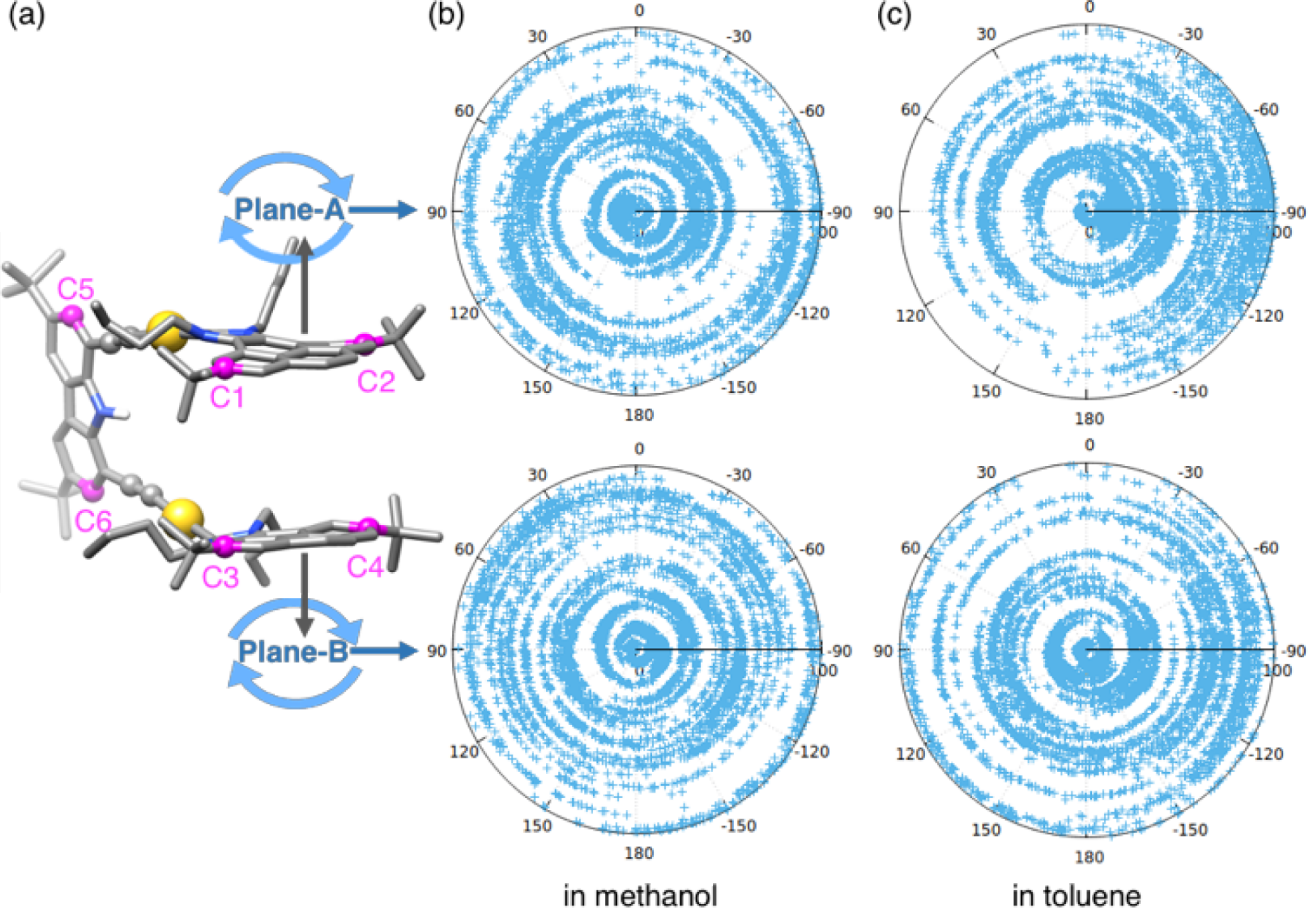
(a) Description of plane A (C1, C2, and C5) and plane B (C3, C4, and C6) of the metallo-tweezers. Dihedral angles between (upper panel) plane A and the carbazole spacer and (lower panel) plane B and the carbazole spacer (b) in methanol and (c) in toluene.

We also performed a probability analysis on the rotation of the two arms of the host; the highest probability is estimated to be less than 5% and 3.5% in toluene and methanol, respectively (Figures S9 and S10), indicating no favored dihedral angles between the arms. The main conclusion of this section is that the Au­(I)-based metallo-tweezer exhibits a very flexible system with no clear preferential geometry. Interestingly, the movement of the pyrene fragments of the metallo-tweezers is similar to the “ballbot-type” motion described for N-heterocyclic carbenes (NHCs) on gold surfaces, which indicates free rotation around a single bond between gold and the NHC carbon atom.^75^ Moreover, this flexibility does not seem to be affected by the nature of the solvent, as the behavior is very similar when two very different solvents, such as MeOH or toluene, are used. Therefore, on this basis, there is no preorganization of the system without the ligand. Furthermore, we analyzed the rotations of the host arms, and no clear correlation was found in their relative motion (see Figure S11).

### Characterization of Host–Guest Complexes

Once the behavior of the unbound metallo-tweezer in both solvents was characterized, we turned our attention to the host–guest complexes. Experimentally, the molecular recognition process of several polycyclic aromatic hydrocarbons (PAHs) and their functionalized forms was investigated in toluene.^44^ The guests functionalized with hydrogen-bond-accepting groups have higher binding affinities compared to the corresponding unfunctionalized forms, as a consequence of the H-bonding interaction with the N–H group of the carbazolyl linker, according to previously reported work.^44^ The largest difference in binding affinity between the functionalized and unfunctionalized forms was measured for pyrene and its functionalized form, 1-pyrene-methanol (34× larger for the latter). We thus focused on these two guests in the present study.

We analyzed the movement of pyrene-imidazolylidene-based planes A and B of the metallo-tweezers for each host–guest complex. The results showed that complete rotations of each plane were observed for pyrene as a guest, whereas such a rotation was never observed for 1-pyrene-methanol, at least not within the limited 100 ns time scale covered in the simulations (Figure 3). This result clearly demonstrates that the presence of the hydroxyl functional group significantly influences the stability and configuration of the complexes, aligning at least qualitatively with the experimental trends in ligand binding. The dynamics of the metallo-tweezers are significantly altered by the presence of the guest, and the nature of the guest also affects the dynamic behavior, especially in 1-pyrene-methanol.

**Figure 3:**
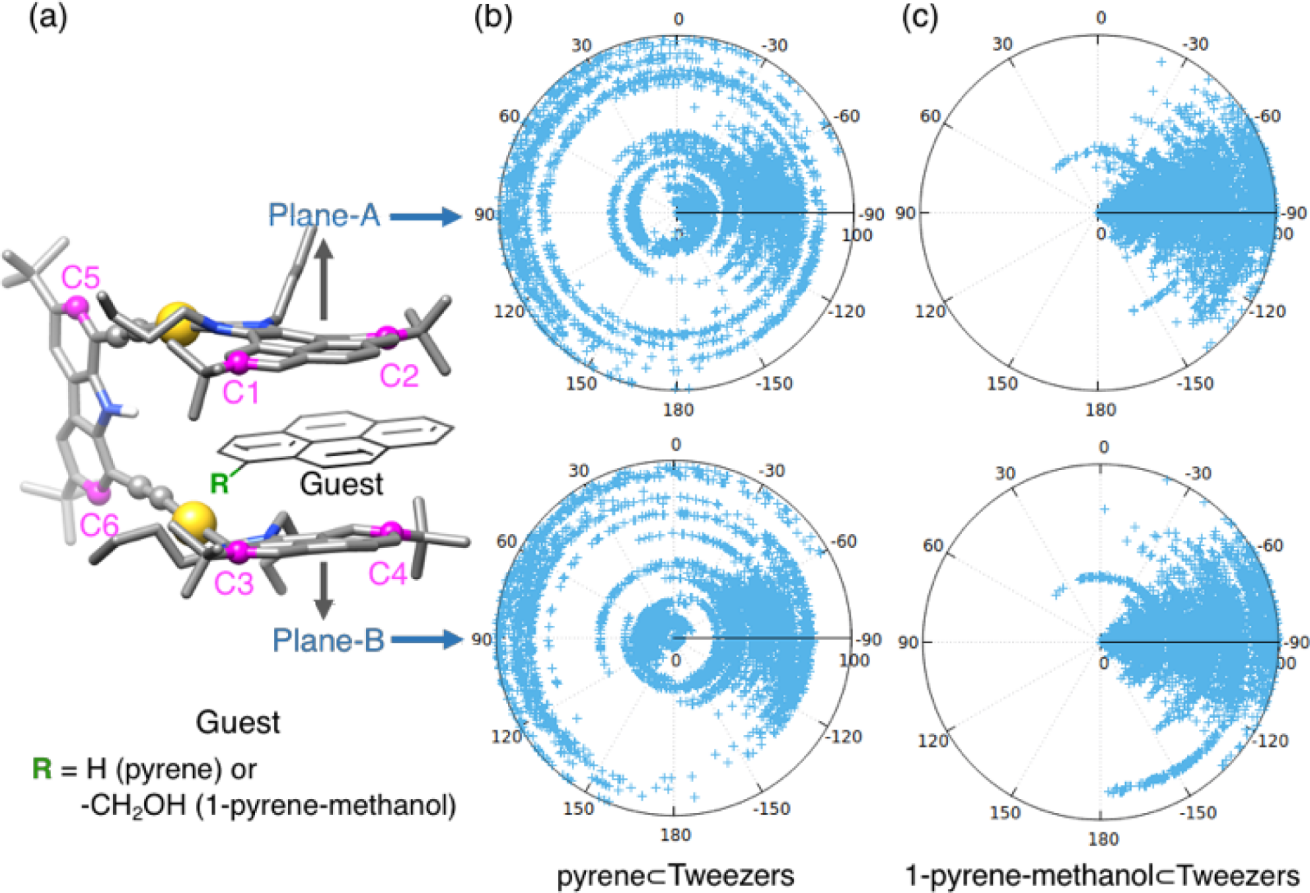
(a) Description of planes A and B defined by two carbon atoms selected on each pyrene fragment of the metallo-tweezers. Dihedral angles between (upper panel) plane A and the carbazole spacer and (lower panel) plane B and the carbazole spacer (b) for pyrene and (c) for 1-pyrene-methanol.

Next, we analyzed the behavior of the guest and whether it remained between the two arms of the metallo-tweezers during the simulation. We first established a reference point defined by the center of mass (COM) between the two polyaromatic hands (Figure 4). We then monitored the evolution of the distance between the COM and the guest. Interestingly, the pyrene guest was located between the host’s pyrene arms in the most populated structure of the host–guest complex. In addition, the analysis along the MD simulation shows that the pyrene guest is also reversibly released from the cavity during the simulation. This leaves a situation in which the host is able to fully rotate its polyaromatic arms when the guest is out of the cavity (Figure 3). On the contrary, 1-pyrene-methanol remained between the two aromatic arms during the whole simulation, thus generating a more rigid structure in which the host’s polyaromatic hands are unable to rotate. These results are in full agreement with the experimental findings, which showed a stronger binding affinity for 1-pyrene-methanol than for pyrene.

**Figure 4:**
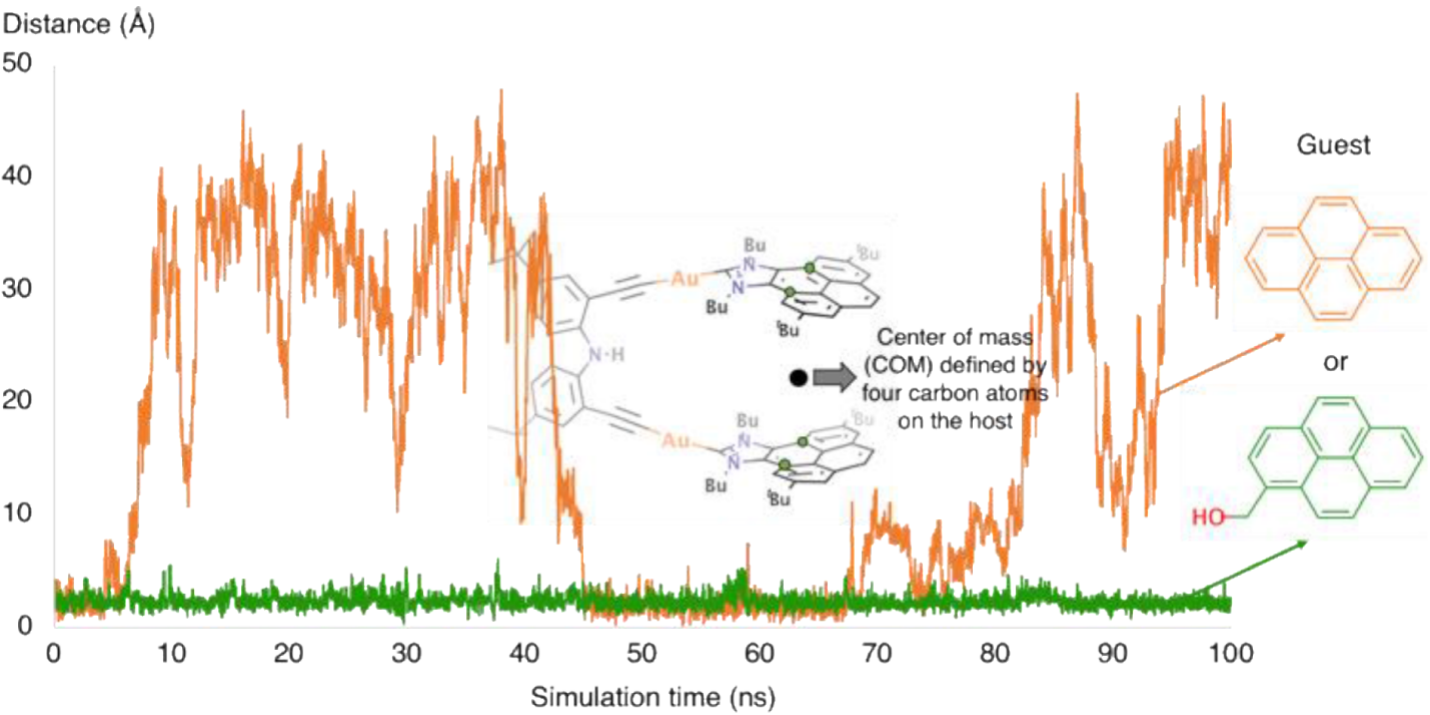
Distance between the center of mass (COM) of the guest and the COM defined by the selected atoms on each pyrene fragment of the metallo-tweezers.

Further analysis of the MD simulations of each host–guest complex in toluene was pursued in order to shed more light on the different binding behaviors of both guests. We looked at the most populated clusters (26% and 12% for pyrene and 1-pyrene-methanol, respectively) with sandwiched conformations in detail. For both structures, the guests are bound between the two pyrene fragments of the host, and the orientation of the aromatic rings of the guests is quite similar (Figure 5 Top view). The distances between the aromatic rings of the guest and the two pyrene units of the host are also similar for both structures: 3.51 (3.85 ± 0.34) and 3.29 (3.88 ± 0.35) Å for pyrene, and 3.52 (3.73 ± 0.35) and 3.37 (3.77 ± 0.29) Å for 1-pyrene-methanol (Figure 5 Front view). Interestingly, there are some differences in the relative arrangements of the aromatic groups between them. While for the nonfunctionalized guest species the packing between the three aromatic moieties is highly symmetrical, for the functionalized guest, a sliding can be observed (Figure 5).

**Figure 5:**
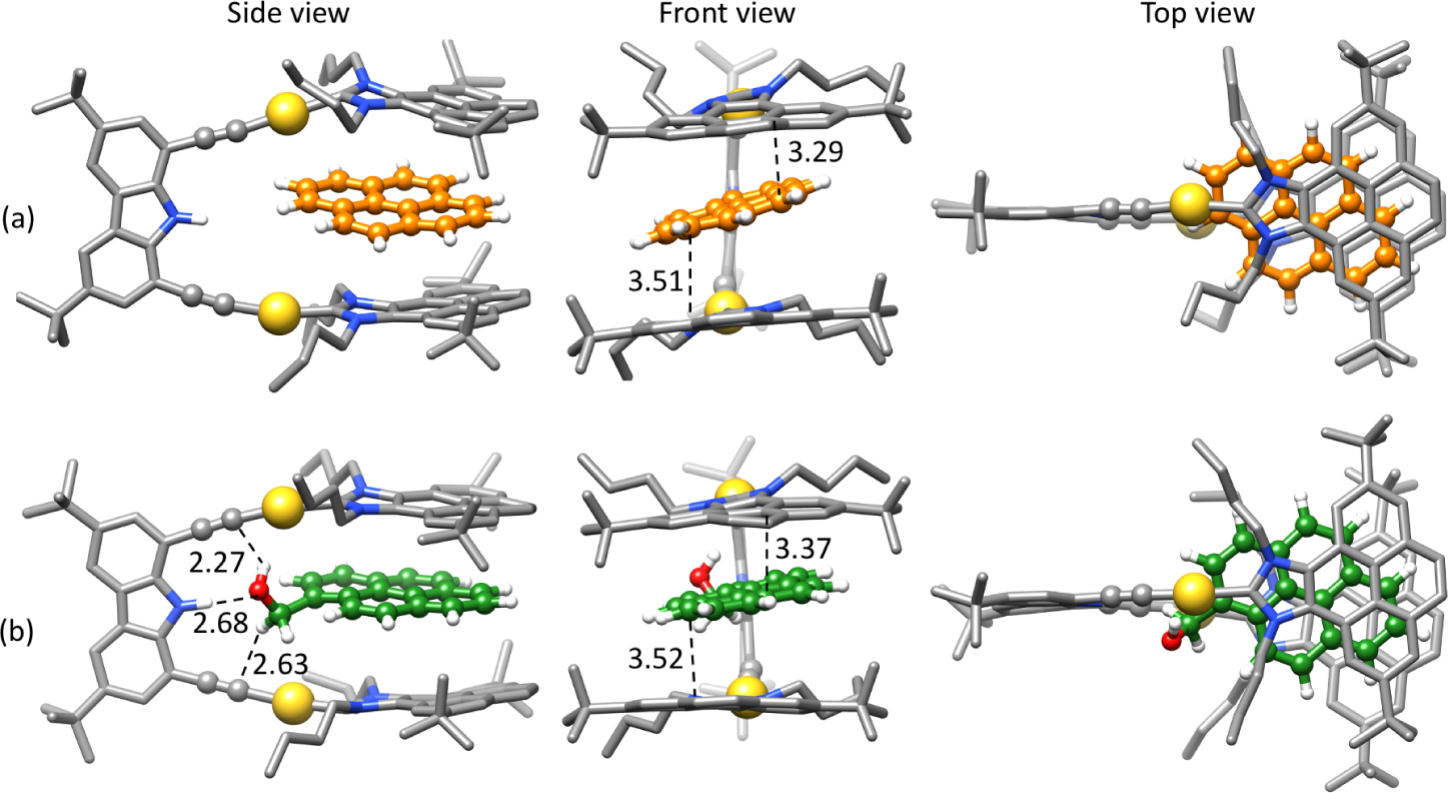
Most populated structures obtained from clustering the MD simulation of each host–guest complex in toluene. (a) Pyrene and (b) 1-pyrene-methanol as the guest molecule. The hydrogens of the metallo-tweezers are omitted for clarity. Distances are given in Å.

This correlates well with an apparent H-bonding interaction between the OH group of the guest and the central NH group of the host. The possibility of a direct hydrogen bond between both moieties was initially proposed based on X-ray data and DFT calculations.^44^ When analyzing the average structure along the MD simulation, however, the location of the guest inside the tweezer is quite symmetrical for both guests. For pyrene, the distances to the aromatic arms are 3.85 ± 0.34 and 3.88 ± 0.35 Å, respectively. The analogous distances for 1-pyrene-methanol are 3.73 ± 0.35 and 3.77 ± 0.29 Å, respectively.

Further analysis of the MD simulation regarding the distance between H and O (—NH···OH—, distance d1 in Figure 6) shows that it fluctuates widely between 2 and 8 Å, with an average value of 3.6 Å.

**Figure 6:**
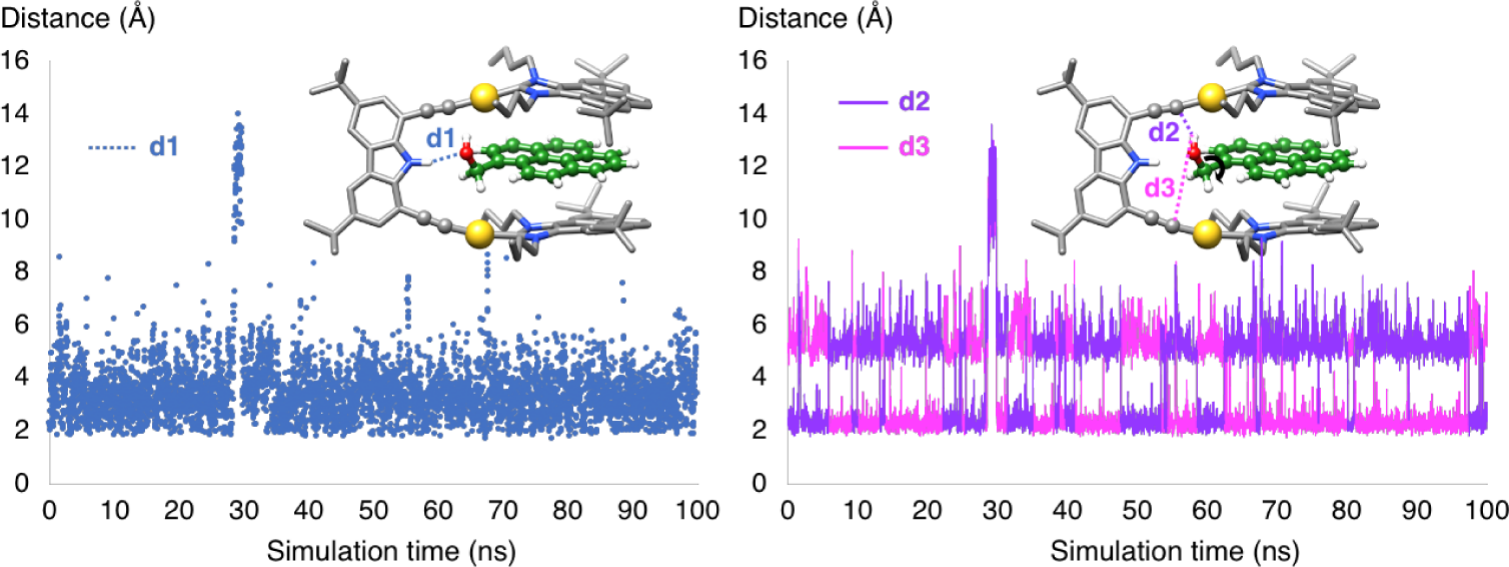
Key distances between the functional group of the guest (1-pyrene-methanol) and the host: d1 corresponds to the distance between the hydrogen of the hydroxyl group of the guest and the nitrogen of the NH of the metallo-tweezer; d2 and d3 correspond to the distances between the hydrogen of the hydroxyl group of the guest and the carbon atom of the alkynyl moieties.

This corresponds to a relatively weak interaction. Interestingly, while the H-bonding interaction term of this nature is not explicitly introduced in the AMBER force field, the analysis of the simulations shows that it is effectively captured through the Lennard-Jones potential and Coulomb terms. However, the polar component of the interaction does appear in another unanticipated fashion. Indeed, we identified an interaction between the hydroxyl moiety of the guest and the alkynyl groups of the host that is present in the MD simulation. This interaction switches constantly from one arm to another, but in both situations, the —OH···(—C≡C—) distances (d2 and d3) when interacting range from 2 to 3 Å. The simulation showed that the OH group of 1-pyrene-methanol is pointing alternately toward one of the two alkynyl groups of the host. The only exception appears for 2 ns during a total simulation time of 100 ns (28–30 ns, Figure 6).

The simulations clearly show the prevalence of the polar interaction between the −CH_2_OH group and the alkynyl moieties over the NH fragments of the host; this can substantially contribute to the higher affinity of the functionalized guest. To better evaluate the nature of this interaction and its intensity, we selected a series of snapshots and optimized their geometries using full-DFT calculations. In all cases, the —OH···(—C≡C—Au) interaction is present in the optimized structures, with distances rounding 2.5 Å from carbon to gold. Similar distances are found for the —OH···(—C≡C—) interaction in the literature.^76^ On the contrary, not all of the complexes maintain the NH···OH contact, showing that the former is more discriminative than the latter The relevance of the two types of polar interactions was further analyzed by calculating their relative Gibbs energies and by comparing structural differences and the overall π-stacking interactions by means of NCI analysis. Differences in Gibbs energies show that both structures are mostly isoenergetic (0.4 kcal/mol), with the geometry with the —NH···OH— hydrogen bond being less favorable. This indicates that both interactions contribute to the stability of the host–guest complex. The results agree well with the MD occupancy, which shows both arrangements with a preference for the latter and gives validity to the force field developed for the system. Indeed, by forming the H-bond, the linker and the pyrene need to adapt to maintain most of the π-stacking. This produces a deviation of the carbazole from orthogonality with respect to the pyrenes of the arms and forces one of them to deviate from a parallel interaction with the guest (from 88° in one conformation to 49° in the other, Figure 7b). Such a balance between polar interactions, π-stacking, and the shape of the molecule can be better understood when considering NCI analysis. The structure with the —OH···(—C≡C—Au) interaction presents a well-organized and parallel geometry with mostly attractive NCI patterns. On the contrary, the structure with the NH···OH hydrogen bond partially loses the packing. In this latter case, small repulsive interactions are more prevalent, mainly for the *n*-butyl group of the pyrene arm at the closest distance to the ipso carbon of the guest (orange area in Figure 7b right). This clearly illustrates the fine-tuning of the different forces in these systems.

**Figure 7:**
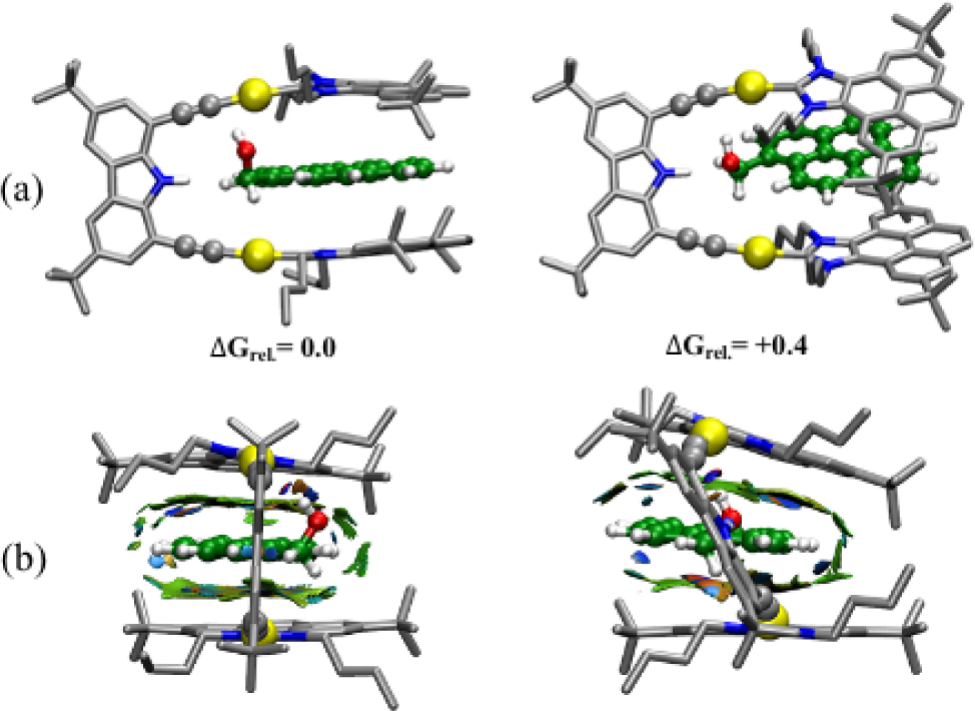
DFT-optimized structures of the two lowest energy conformations with NH···OH (right side) and with the OH····(C≡C–Au) interaction (left side). (a) Side view of the optimized structure and (b) front view of the NCI representation with the carbazole facing the reader; pyrene-carbazole dihedral angles are 88° and 49°, respectively. NCI surfaces correspond to *s* = 0.3 au and a color scale of −1.5 < ρ < 1.5 au for promolecular densities. Gibbs energies are given in kcal·mol^–1^.

### Binding of the Guests to the Metallo-Tweezers

The results presented above suggest that, in addition to π-stacking and hydrogen bonding, the attractive electrostatic interaction between the hydroxyl group of the guest and the alkynyl groups of the host plays a significant role in the higher binding affinity of 1-pyrene-methanol to the metallo-tweezers compared to that shown by pyrene. To evaluate the energetics and gain a more accurate understanding of the binding process, we performed metadynamics simulations. Since we have the experimental binding constants for both guests, we focused on obtaining the thermodynamics of the binding process. Note that standard well-tempered metadynamics simulations do not necessarily yield reliable absolute kinetics without dedicated formalisms.^77^ The distance between the center of mass (COM) of the guest and the nitrogen atom of the NH group of the host was chosen as the collective variable (CV). The calculated binding Gibbs energies are 3.8 and 4.1 kcal/mol for pyrene and 1-pyrene-methanol, respectively (Figure 8). These values show very good agreement with the experimental values of 1.4 and 3.5 kcal/mol, respectively. Another metadynamics simulation with 1 collective variable, which is the distance between the COM of the guest and the COM defined by four selected atoms from the host’s arms (two from each pyrene arm), was also performed for comparison, obtaining similar results; the computed free energy profiles are provided in the Supporting Information (Figure S1). Moreover, we performed metadynamics simulations with 2 collective variables (selecting as the second collective variable a dihedral angle for the arm rotation); the 2D surfaces obtained are given in the Supporting Information (Figure S12). The free energy surface (FES) shows two minima at around ± 90° for the encapsulated guest; a pathway for arm rotation that maintains the guest at ∼6–8 Å is the most feasible one. This suggests that the guest remains π-stacked to the arm during the flip, supporting this process as an encapsulation mechanism. Note that direct encapsulation without an arm flip cannot be ruled out. The FES obtained from all of these simulations are similar in both surface shapes and energy values.

**Figure 8:**
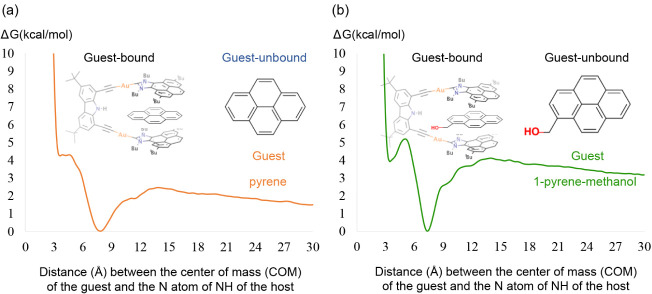
Gibbs energy profiles for the binding of (a) pyrene and (b) 1-pyrene-methanol to the metallo-tweezers.

We found that the interaction between the hydroxyl group of the guest and an alkynyl group from one of the host’s arms helps stabilize the host–guest complex in this region. This attractive interaction may, therefore, serve as one of the driving forces for the formation of the complex. Additionally, structures in which the host and guest interact through π-stacking, with the guest located outside the host, are also observed in this region, suggesting that these interactions also play a significant role in stabilizing the complex. Importantly, we identified a pathway for encapsulation in which the guest initially forms a π-stacking interaction with the external side of one of the polyaromatic arms of the host. This is followed by a rotation of the polyaromatic arm, allowing the guest to be accommodated inside the cavity of the metallo-tweezer (Figure 9). This observation indicates that the guest’s approach to the metallo-tweezer is more favorable via π-stacking interactions with the outer part of the tweezer than by directly entering the space between the arms.

**Figure 9:**
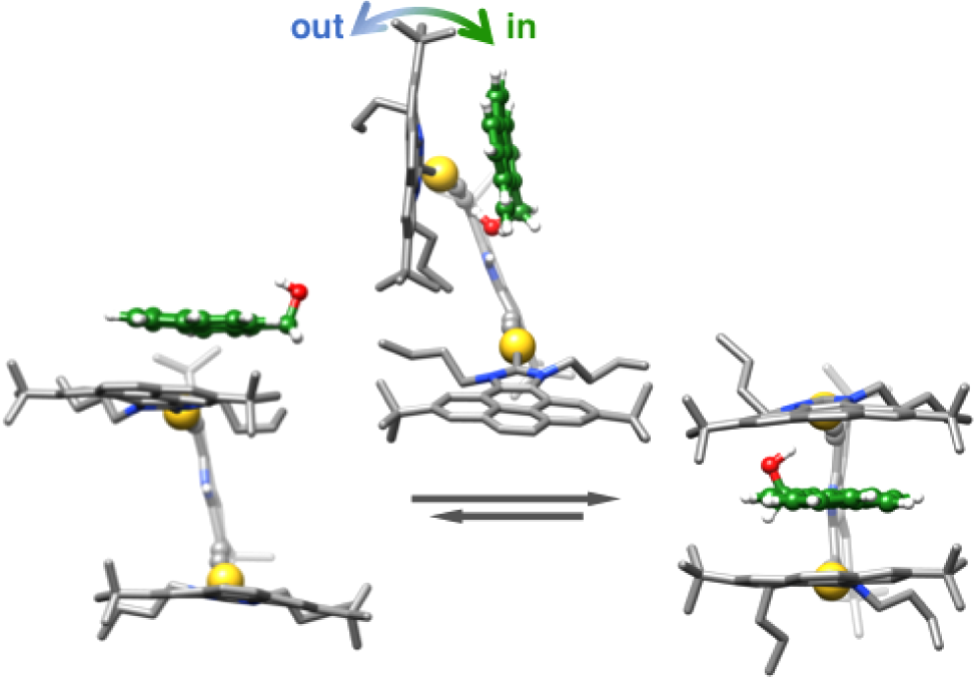
Binding mechanism of the guest to the metallo-tweezers.

## Conclusions

The complete set of simulations and their analyses reveals a novel and unforeseen mechanism for guest recruitment. Collectively, the calculations demonstrate that, in the absence of a guest, the arms of the metallo-tweezer undergo spontaneous free rotation, allowing for significant variability in the accessible space between the two pyrene units. Guest encapsulation is initiated through a strong aromatic interaction between the polyaromatic guest and the exposed face of one of the pyrene units of the metallo-tweezer. This interaction triggers the flipping of the pyrene-imidazolylidene ligand around the Au–C bond, effectively sandwiching the guest between the two pyrene units of the tweezer. This mechanism underscores the pivotal role of π-stacking in the recognition and encapsulation of the guest. Furthermore, the study highlights that, while π-stacking is central to the encapsulation process, polar interactions are crucial in enhancing the binding affinity observed with functionalized guests, thereby driving stronger interactions in the system.

## Supplementary Material



## Notes

Additional analysis of metadynamics simulations and Cartesian coordinates for optimized structures. The input/output files for Gaussian, AMBER, and GROMACS/PLUMED are available at the CORA repository: 10.34810/data2452 (PDF)

## Abbreviations

B3LYP-D3: dispersion-corrected Becke three-parameter Lee–Yang–Parr exchange–correlation functional
SMD: solvent model density
quasi-RRHO: quasi-rigid-rotor-harmonic-oscillator approximation
MD: molecular dynamics
MCPB.py: Python-based metal center parameter builder program
RESP: restrained electrostatic potential method
UFF: universal force field
WT-MetaD: well-tempered metadynamics
CV: collective variable
COM: center of mass
