# Supplementary material for: Guest Binding Mechanism of Polycyclic Aromatic Hydrocarbons by Au(I) Metallo-Tweezers Revealed by Computation
Source: Inorg Chem. 2025 Nov 13;64(46):22664–73. doi: 10.1021/acs.inorgchem.5c03400 (PMC12648653; doi:10.1021/acs.inorgchem.5c03400)
Supplement: Supplementary file 1 [file ic5c03400_si_001.pdf]

Guest binding mechanism of polycyclic aromatic hydrocarbons by Au(I) metallo-tweezers revealed by computation

Gantulga Norjmaa,[a] Susana Ibáñez,[b] Eduardo Peris, *[b] Jean-Didier Maréchal*[a] and Gregori Ujaque*[a]

[a]*Departament de Química, Universitat Autònoma de Barcelona, 08193 Cerdanyola del Vallès, Catalonia, and Centro de Innovación en Química Avanzada (ORFEO-CINQA), Catalonia*
[b]*Institute of Advanced Materials (INAM), Universitat Jaume I, Av. Vicente Sos Baynat s/n, Castellón, 12071 (Spain)*

Emails: eperis@uji.es jeandidier.marechal@uab.cat gregori.ujaque@uab.cat

Table of contents

1. WT-MetaD simulations with 1 CV. ……………………………………………………..S2

2. Analysis of metadynamics simulations. ……………………………………………………S2

3. Potential energy surface (PES) for the rotation of the host arm. …………………………...S5

4. Analysis on the rotation of the host arms. …………………………………………………S6

5. WT-MetaD simulations with 2 CV. ……………………………………………………..S8

6. References. ………………………………………………..……………………..…S8

7. Optimized cartesian coordinates. ………………………………………………………..S9

1. WT-MetaD simulations with 1 CV.

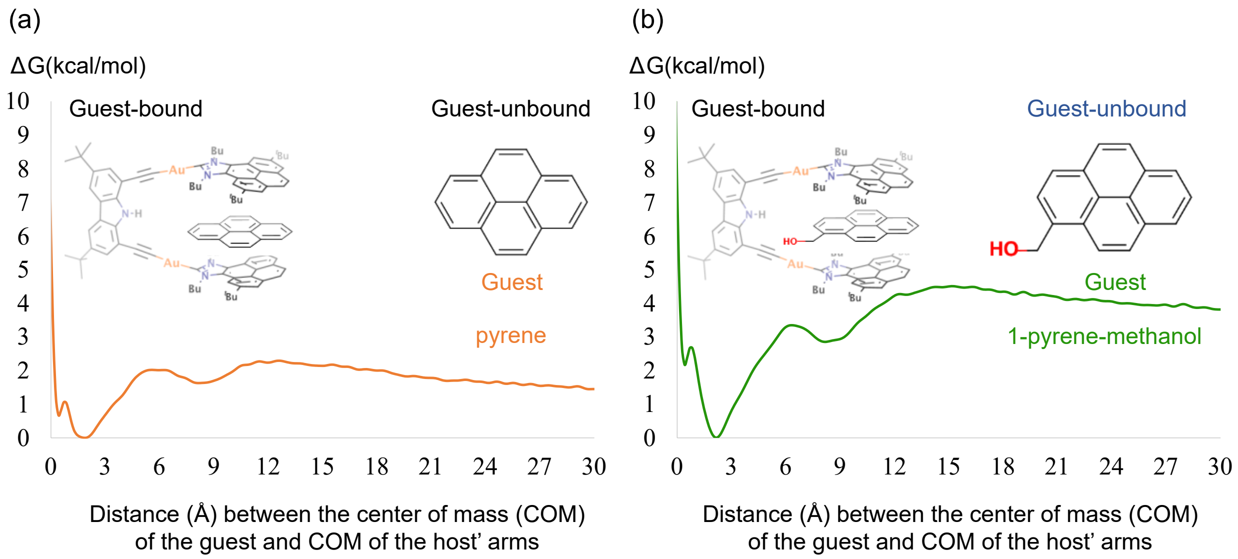

Figure S1. Free energy profiles obtained from metadynamics simulations for binding of (a) 1-pyrenemethanol and (b) pyrene to the metallotweezer. The collective variable of the simulation is a distance between the COM of the two center carbon atoms of each pyrene arms of the host and COM of the guest.

2. Analysis of metadynamics simulations.

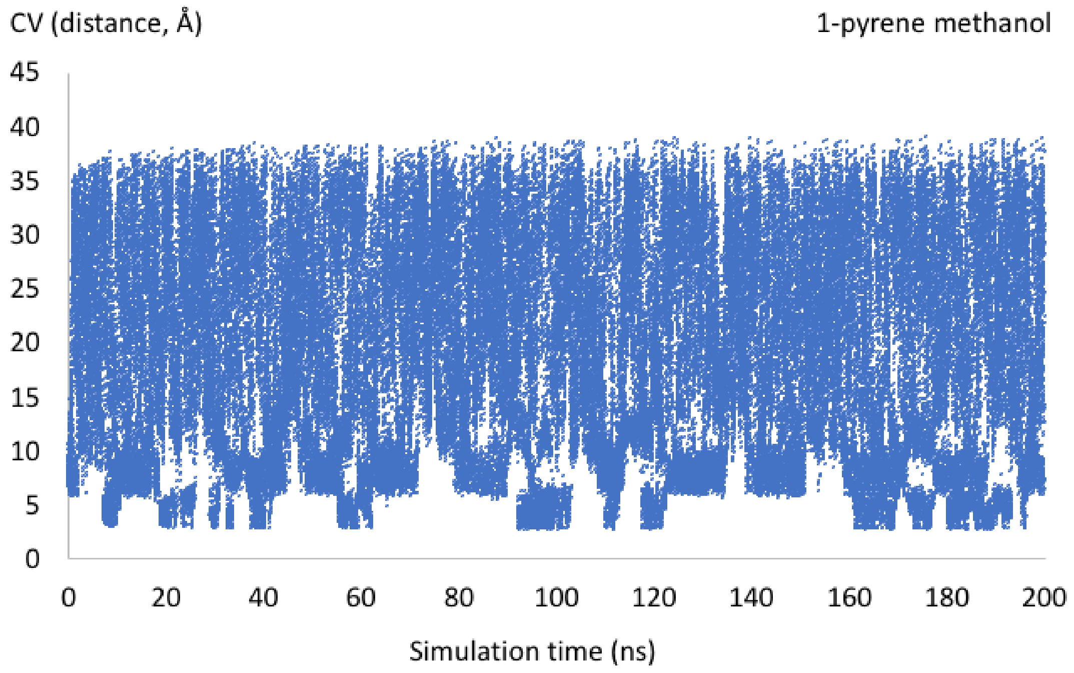

Figure S2. Evolution of the collective variable (distance between N and the COM of the guest) during the simulation of 1-pyrenemethanol.

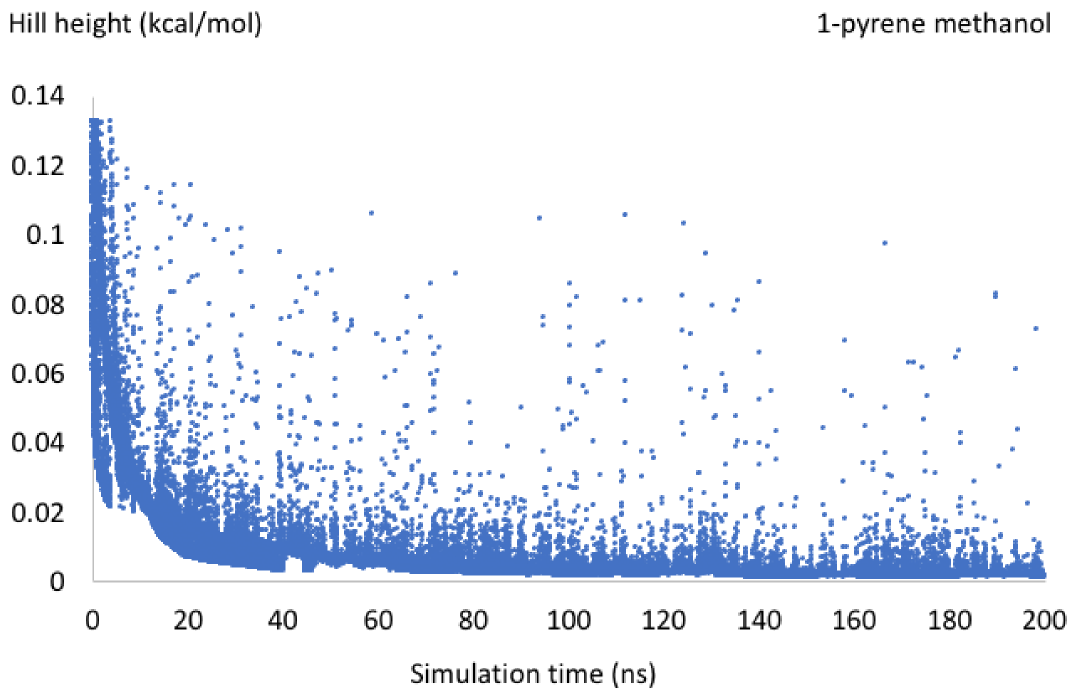

Figure S3. The height of the gaussian hills during the WT-metaD simulation of 1-pyrenemethanol.

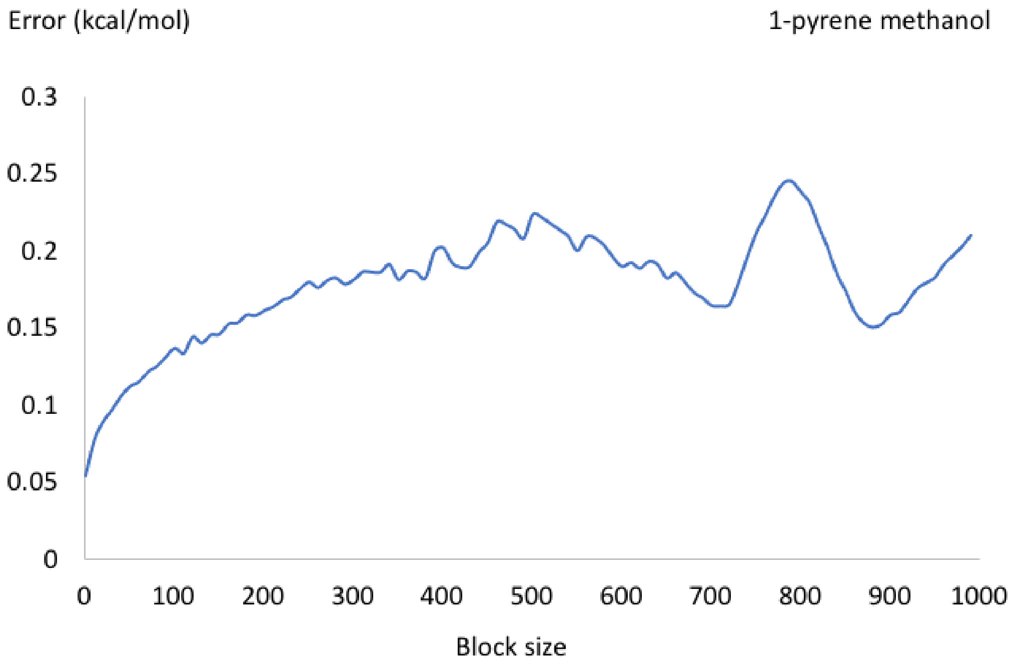

Figure S4. The average error along the block size for the simulation of 1-pyrenemethanol.[1,2]

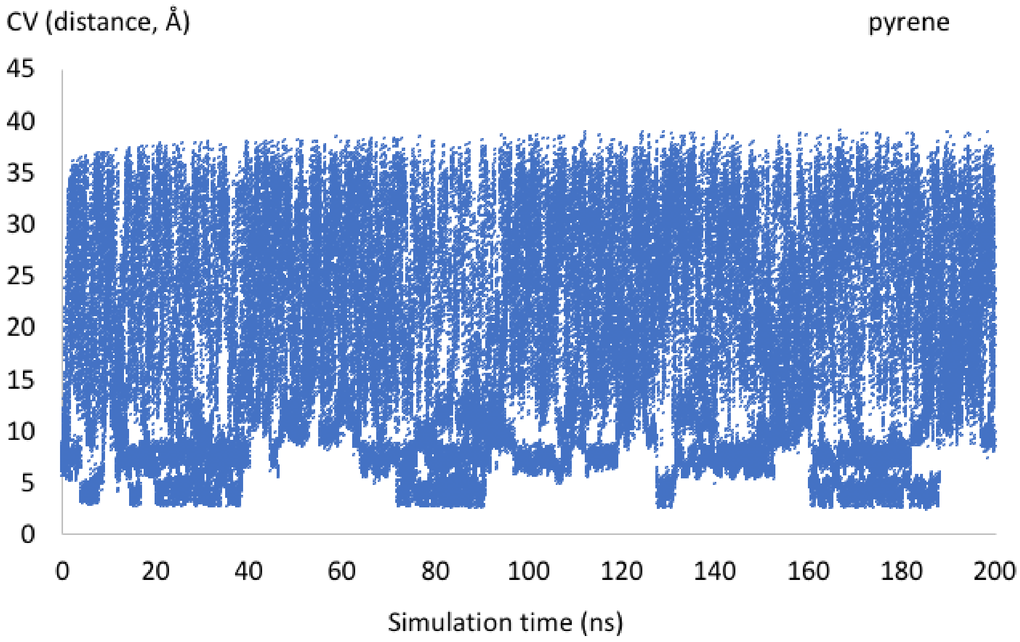

Figure S5. Evolution of the collective variable (distance between N and the COM of the guest) during the simulation of pyrene.

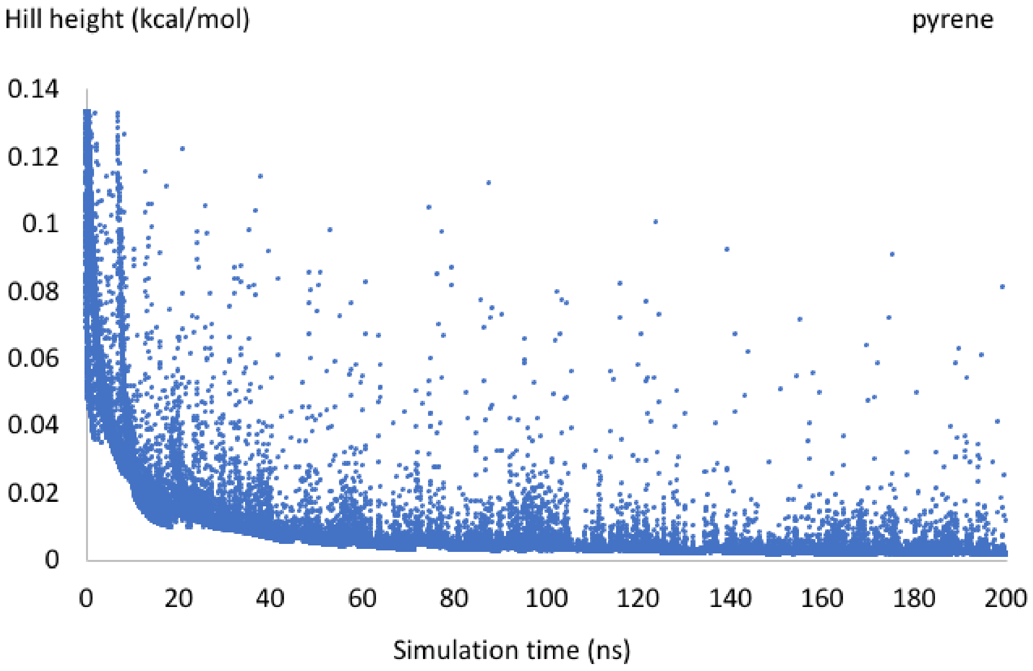

Figure S6. The height of the gaussian hills during the WT-metaD simulation of pyrene.

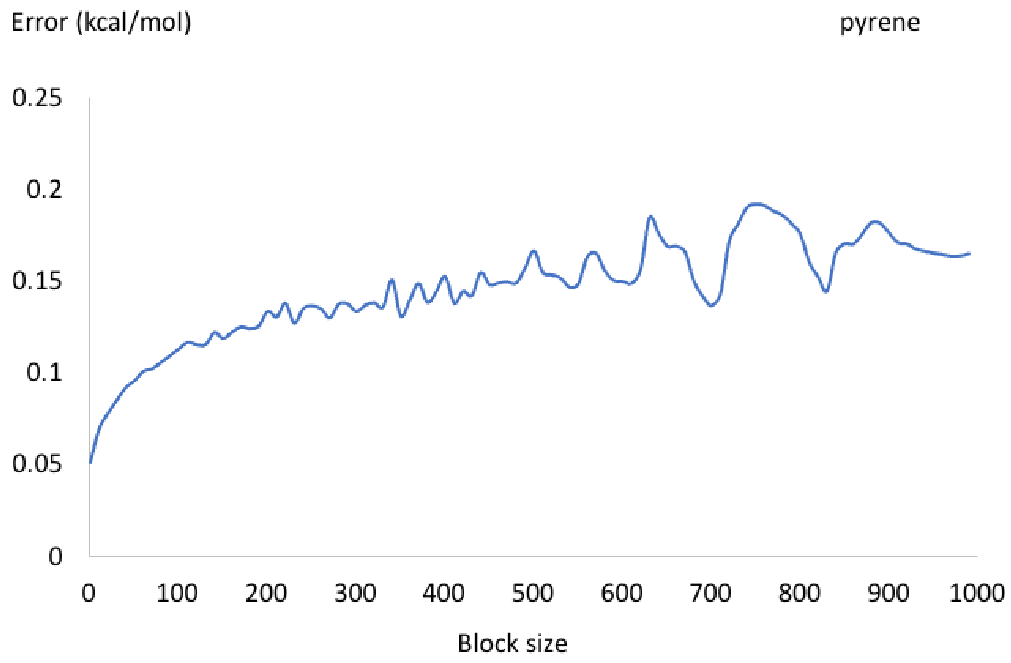

Figure S7. The average error along the block size for the simulation of pyrene.[1,2]

3. Potential energy surface (PES) for the rotation of the host arm.

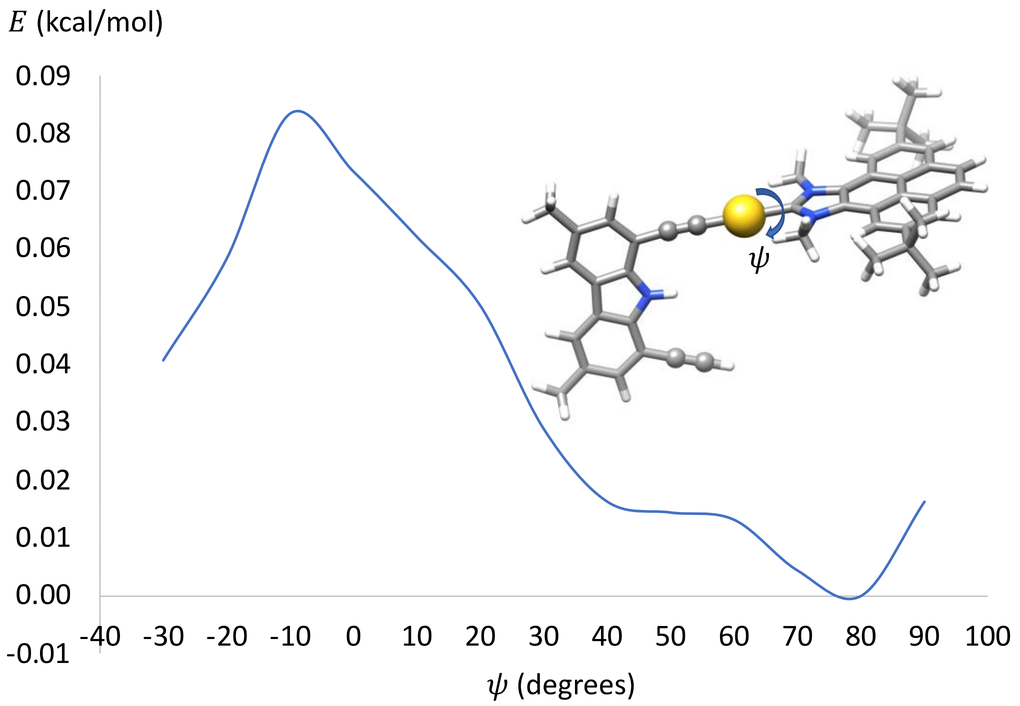

Figure S8. Potential energy surface (PES) for the rotation of the host arm calculated with a reduced model at DFT level; single point calculations every 10 degrees.

4. Analysis on the rotation of the host arms.

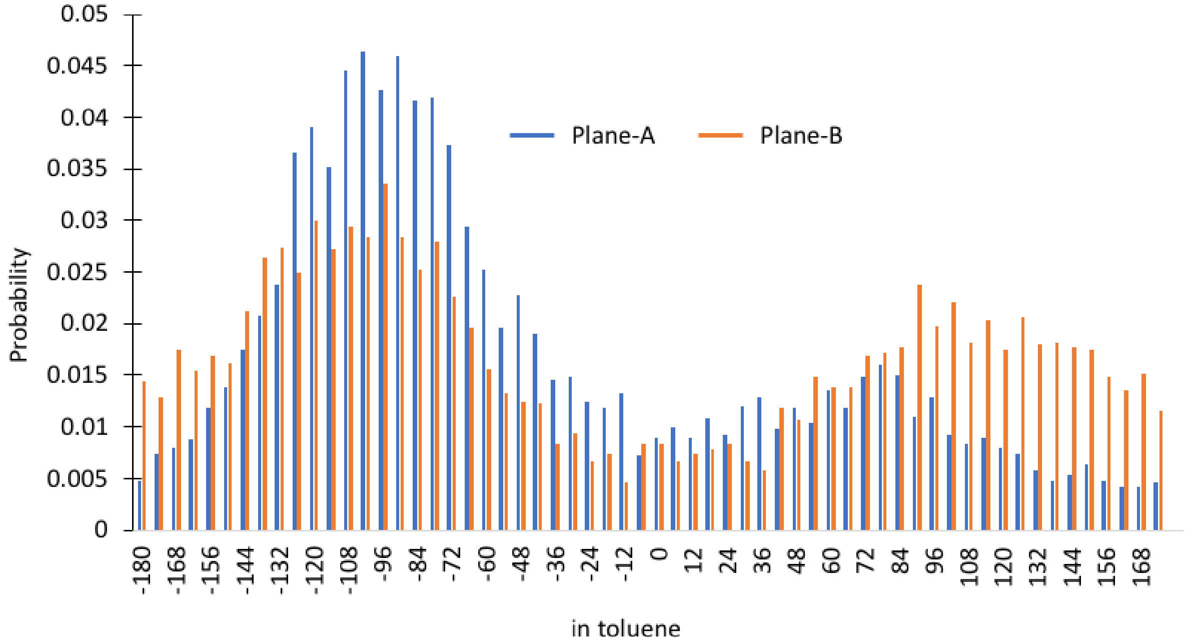

Figure S9. Probability distributions of the dihedral angles of the host arms (Plane-A and Plane-B) relative to the carbazole spacer of the host in toluene.

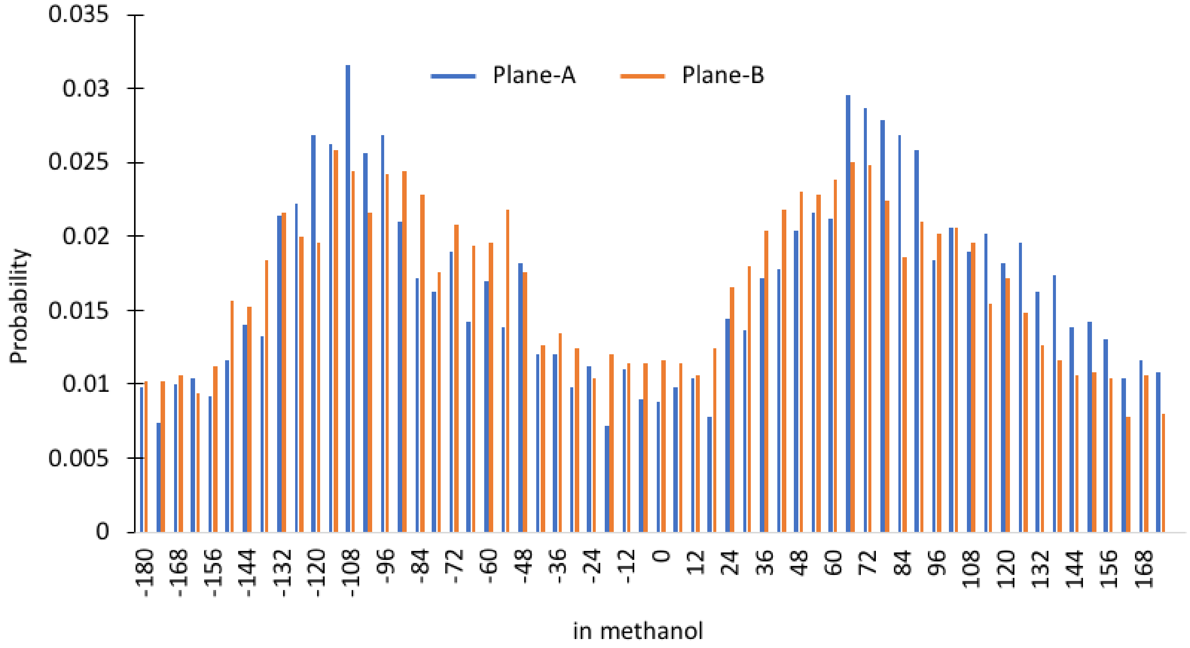

Figure S10. Probability distributions of the dihedral angles of the host arms (Plane-A and Plane-B) relative to the carbazole spacer of the host in methanol.

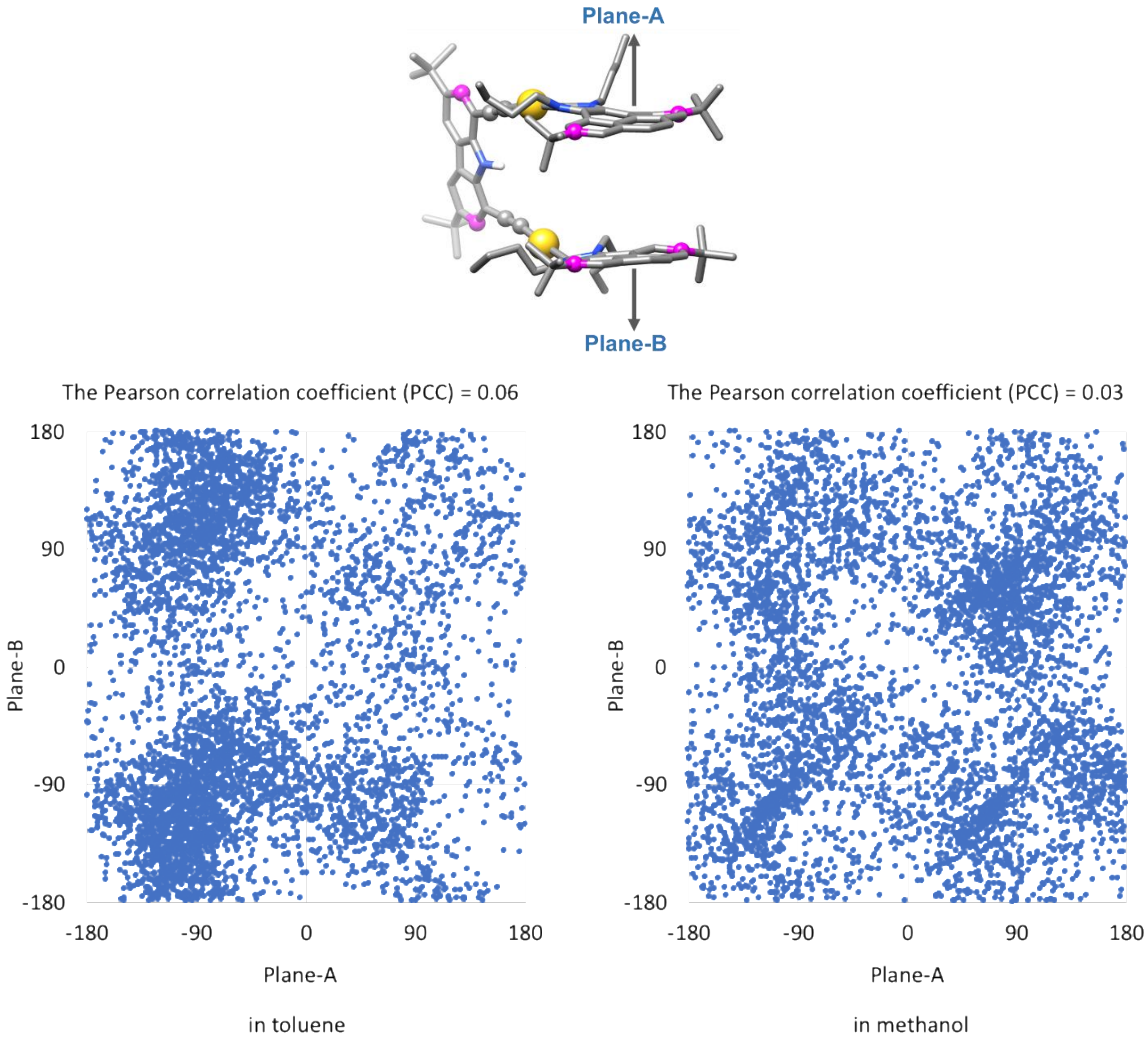

Figure S11. The graphical representation of Plane-A *vs* Plane-B for the correlation analysis on the rotation of the host arms.

5. WT-MetaD simulations with 2 CV.

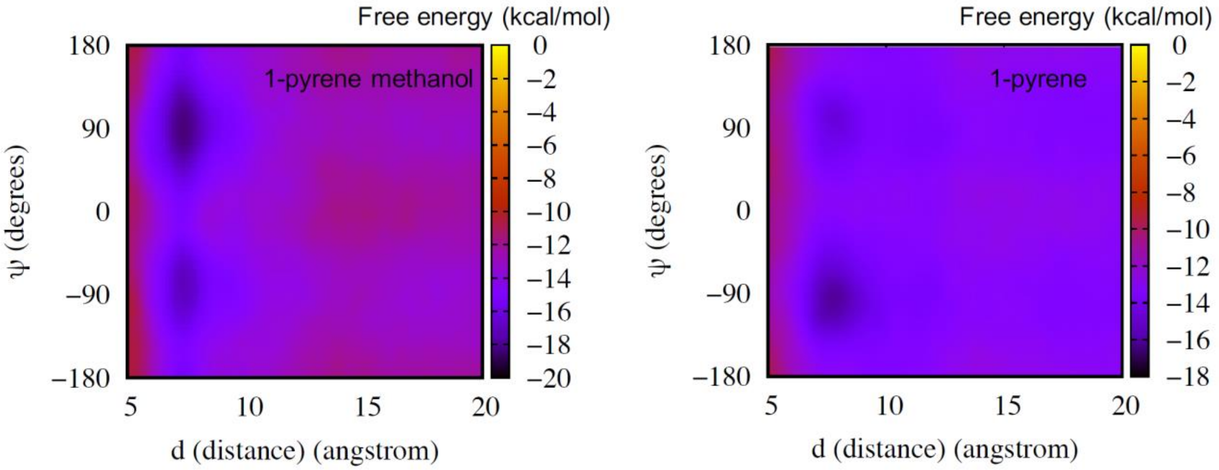

Figure S12. 2D surfaces obtained from metadynamics simulations with 2 CV (one is distance between the host and guest, and the other is dihedral angle for the arm-rotation of the host).

6. References

1. Flyvbjerg, H.; Petersen, H. G. Error estimates on averages of correlated data. *J. Chem. Phys*, **1989**, *91*, 461-466.

2. Grossfield, A.; Zuckerman, D. M. Quantifying uncertainty and sampling quality in biomolecular simulations. *Annu Rep Comput Chem.* **2009**, *5*, 23-48.

7. Optimized cartesian coordinates.

```
204 (tweezer)
scf done: -4039.881493
Au      2.117787     2.914124     1.661007
Au      1.376407    -3.149106    -1.521589
 C     -0.679526    -3.055299    -1.616264
 C      3.356859    -3.049933    -1.359105
 C      0.069694     2.969738     1.827079
 C      4.060286     2.641364     1.338961
 N     -0.720806     1.924275     2.192285
 N     -1.597857    -3.827114    -0.976220
 N     -1.372153    -2.017928    -2.157782
 N     -0.760353     3.907417     1.295994
 C     -3.281234     4.022856     0.734670
 N      5.828484    -0.375005    -0.110419
 C      5.223567     2.309970     1.119524
 C     -3.311327     0.162835     2.728934
 C     -5.696995     3.755654     0.370323
 C     -6.539475    -3.183317    -0.331278
 C     -1.194338    -4.973374    -0.150525
 C      8.057054     0.059352     0.077179
 C     -3.710146     0.068146    -2.682522
 C     -5.073191    -1.556558    -1.479265
 C     -5.249015    -2.811290    -0.811278
 C     -5.681081     0.027721     2.409115
 C     -4.159688    -3.723159    -0.616980
 C     -2.075301     3.435600     1.263495
 C      9.170235     0.833062     0.412627
 C     -4.512397    -0.520694     2.944906
 C     -5.676360     1.242549     1.711416
 C     -4.795532     0.949168    -2.820444
 C     -6.900727     2.983392     0.524312
 C     -6.016839     0.566660    -2.272973
 C     -2.874114    -3.266146    -1.088983
 C     -1.293190    -4.669311     1.346349
 C     -2.721977    -2.091291    -1.812505
 C     -0.193505     0.755298     2.916763
 C     -3.248174     1.394488     2.056160
 C     -3.808426    -1.163922    -2.027549
 C     -4.469216     3.239358     0.888226
 C     -6.891262     1.787207     1.168611
 C     -3.357963     5.256285     0.064499
 C      7.889925    -1.260703    -0.502437
 C     -7.628259    -2.257809    -0.472831
 C      8.790132    -2.244766    -0.928676
 C     -0.271905     5.258117     0.965960
 C     -4.455569     1.965808     1.544173
 C     -4.410792    -4.959472    -0.011512
 C      8.314193    -3.448195    -1.459734
 C     -2.052917     2.176087     1.852527
 C     -0.707734    -1.011805    -2.998444
 C      6.543956     1.837795     0.868127
 C      4.571560    -2.890344    -1.256779
```

C	-4.555769	5.759270	-0.455002
C	-4.574836	-1.800380	3.796849
C	6.918049	-3.633179	-1.556889
C	6.495129	-1.480239	-0.604577
C	-6.729522	-4.434288	0.284460
C	7.696314	2.583631	1.184801
C	-6.178058	-0.665095	-1.612819
C	6.752485	0.566466	0.301692
C	-7.452683	-1.050777	-1.076735
C	-5.858789	-6.718936	1.104683
C	-0.065551	-0.511236	2.050529
C	-5.683961	-5.339599	0.445716
C	10.249463	2.948432	1.321725
C	9.004232	2.111343	0.967668
C	-5.713540	4.994509	-0.284318
C	9.251092	-4.570663	-1.945257
C	0.112461	5.438796	-0.501685
C	-4.622338	7.094902	-1.216926
C	5.976487	-2.677521	-1.140208
C	-4.588679	2.267769	-3.585572
C	1.260819	-0.620472	1.284399
C	-3.443550	3.073334	-2.931025
C	0.375808	1.268946	-3.128192
C	10.736694	-4.222956	-1.737921
C	0.517259	6.883886	-0.812787
C	-0.263142	0.220618	-2.212885
C	-0.885772	-5.871563	2.204625
C	-1.058572	-5.602744	3.701586
C	-4.215972	1.948239	-5.052215
C	2.477932	-0.873401	2.176615
C	-5.855373	3.141678	-3.587993
C	0.867469	7.080344	-2.289852
C	-5.111497	6.832048	-2.659918
C	-3.253873	7.796381	-1.296528
C	11.100992	3.170843	0.050725
C	11.095253	2.194609	2.373418
C	-5.308451	-2.917583	3.028601
C	-5.347399	-1.479710	5.097697
C	-3.177841	-2.327397	4.175336
C	-5.611880	8.045762	-0.505747
C	9.889496	4.329299	1.900334
C	9.021870	-4.817189	-3.454267
C	0.751508	2.536951	-2.363448
C	8.953453	-5.871755	-1.164501
C	-4.893541	-6.848942	2.306118
C	-7.292351	-6.942187	1.618337
C	-5.534310	-7.819591	0.068879
H	9.318050	4.245632	2.831865
H	10.806701	4.886318	2.126128
H	9.305421	4.928974	1.192613
H	11.438953	2.224499	-0.385298
H	10.528469	3.705000	-0.716752
H	11.993783	3.765365	0.284329
H	11.428130	1.217913	2.005328

H	11.990376	2.773486	2.635820
H	10.518792	2.025603	3.290574
H	11.364018	-5.051446	-2.087778
H	11.030336	-3.329069	-2.300501
H	10.971796	-4.054868	-0.680250
H	7.989345	-5.115441	-3.666208
H	9.232040	-3.910804	-4.034591
H	9.680636	-5.615908	-3.819334
H	7.921475	-6.209095	-1.307839
H	9.616063	-6.680496	-1.498939
H	9.110030	-5.727352	-0.089016
H	4.825366	-0.250760	-0.099325
H	7.535423	3.562517	1.619428
H	10.165207	0.434269	0.235870
H	9.853605	-2.054705	-0.836957
H	6.528108	-4.556710	-1.972575
H	-5.017265	-7.826160	2.788528
H	-3.844249	-6.763260	2.004916
H	-5.090897	-6.073509	3.054661
H	-5.654721	-8.813574	0.517026
H	-6.203498	-7.753966	-0.796864
H	-4.504767	-7.744510	-0.298849
H	-7.574494	-6.196565	2.370947
H	-8.028450	-6.908261	0.806830
H	-7.367065	-7.929471	2.087926
H	-0.741061	-6.464276	4.300237
H	-0.463637	-4.737634	4.019757
H	-2.107571	-5.393691	3.945106
H	-1.488452	-6.745973	1.919300
H	0.159229	-6.133880	1.990660
H	-2.316213	-4.364905	1.598279
H	-0.642731	-3.813211	1.568165
H	-1.793051	-5.842943	-0.432739
H	-0.160005	-5.195208	-0.421425
H	-3.601896	-5.662387	0.108169
H	-7.727068	-4.673734	0.635603
H	-0.135885	2.974246	-1.893318
H	1.477761	2.325494	-1.569149
H	1.188810	3.294017	-3.024708
H	-0.322967	1.523537	-3.939165
H	1.265383	0.839136	-3.608162
H	-1.117069	0.656988	-1.677935
H	0.457715	-0.097588	-1.451393
H	-1.380715	-0.760125	-3.820911
H	0.162270	-1.501840	-3.440558
H	-2.764712	0.375128	-3.096917
H	-6.881554	1.216406	-2.341257
H	-8.283000	-0.356478	-1.176806
H	-8.602511	-2.546183	-0.085674
H	-5.008075	1.372064	-5.544715
H	-4.069019	2.876858	-5.617512
H	-3.288724	1.368526	-5.122829
H	-6.159890	3.410380	-2.571405
H	-5.661017	4.072111	-4.132405

H	-6.695246	2.641173	-4.084086
H	-3.674896	3.304705	-1.888736
H	-2.492858	2.529545	-2.953217
H	-3.290833	4.019190	-3.464699
H	-2.459576	5.829742	-0.080028
H	-6.663625	5.350862	-0.672338
H	-2.513178	7.190652	-1.831470
H	-3.354552	8.743106	-1.838908
H	-2.856786	8.029588	-0.301796
H	-6.104085	6.370290	-2.677856
H	-5.172280	7.774168	-3.218520
H	-4.422186	6.165430	-3.190211
H	-6.622785	7.626671	-0.460230
H	-5.289476	8.254441	0.521204
H	-5.673429	9.000147	-1.042872
H	0.014385	6.832263	-2.934882
H	1.152230	8.118118	-2.498394
H	1.705357	6.437166	-2.585541
H	-0.301626	7.563118	-0.536650
H	1.377137	7.163212	-0.188606
H	-0.720991	5.138685	-1.148959
H	0.945611	4.762975	-0.723157
H	-1.037173	5.971247	1.281094
H	0.603029	5.430034	1.596282
H	-7.806735	1.213690	1.286633
H	-7.824998	3.386389	0.117632
H	-6.631372	-0.482100	2.534806
H	-2.407726	-0.270917	3.115768
H	0.781149	1.055041	3.303389
H	-0.836213	0.598748	3.785258
H	-0.168176	-1.382923	2.711717
H	-0.909048	-0.554752	1.350349
H	2.338004	-1.779283	2.781710
H	3.378601	-1.019404	1.571247
H	2.681208	-0.035906	2.854009
H	1.177035	-1.444291	0.566314
H	1.422581	0.288443	0.690885
H	-6.328142	-2.634342	2.752984
H	-4.776671	-3.177878	2.109855
H	-5.376405	-3.819493	3.648916
H	-5.408939	-2.370039	5.736067
H	-4.848986	-0.686671	5.667490
H	-6.369803	-1.147282	4.885699
H	-3.278409	-3.253676	4.751494
H	-2.576661	-2.553878	3.287633
H	-2.620341	-1.618925	4.799060

****

26 (pyrene)

scf done: -615.794241

C	-4.271178	-5.696066	-0.000605
C	-2.876930	-5.694067	-0.000554
C	-2.161816	-4.485599	-0.000483

```
 C    -2.883174    -3.254509    -0.000434
 C    -4.310022    -3.264374    -0.000505
 C    -4.982642    -4.496961    -0.000582
 C    -0.722285    -4.442800    -0.000463
 C    -2.176522    -2.011701    -0.000275
 C    -0.749718    -2.001789    -0.000210
 C    -0.050287    -3.260765    -0.000331
 C    -0.075512    -0.769723    -0.000011
 C    -0.788572     0.429042     0.000112
 C    -2.182828     0.427663     0.000044
 C    -2.897960    -0.780671    -0.000154
 C    -4.337523    -0.823471    -0.000252
 C    -5.009535    -2.005470    -0.000429
 H    -6.096618    -2.024670    -0.000579
 H    -4.877019     0.120488    -0.000144
 H    -0.182802    -5.386770    -0.000549
 H    -4.808196    -6.640652    -0.000648
 H    -2.331089    -6.634007    -0.000560
 H    -6.069514    -4.508714    -0.000651
 H     1.036839    -3.241932    -0.000325
 H    -0.251676     1.373769     0.000257
 H    -2.728715     1.367604     0.000153
 H     1.011588    -0.756876     0.000061

****************************************

   30 (1pyrenemethanol)
scf done:  -730.316392
 C    -4.271123    -5.696629    -0.000643
 C    -2.877820    -5.694924    -0.000601
 C    -2.162890    -4.485808    -0.000489
 C    -2.878794    -3.251045    -0.000406
 C    -4.307832    -3.262876    -0.000464
 C    -4.979724    -4.495575    -0.000577
 C    -0.728062    -4.442584    -0.000463
 C    -2.170324    -2.010287    -0.000270
 C    -0.740701    -1.995305    -0.000225
 C    -0.051928    -3.260637    -0.000338
 C    -0.060812    -0.755681    -0.000064
 C    -0.793296     0.433096     0.000038
 C    -2.186204     0.422871    -0.000017
 C    -2.899675    -0.782670    -0.000171
 C    -4.337048    -0.825593    -0.000228
 C    -5.009991    -2.007776    -0.000374
 H    -6.096477    -2.025431    -0.000421
 H    -4.877814     0.117025    -0.000147
 H    -0.185437    -5.384189    -0.000535
 H    -4.809184    -6.639870    -0.000705
 H    -2.330596    -6.633724    -0.000645
 H    -6.066314    -4.504483    -0.000601
 H     1.032896    -3.274137    -0.000319
 H    -0.257794     1.373376     0.000171
 H    -2.733187     1.361940     0.000075
 C     1.455584    -0.724986    -0.000002
```

H	1.829307	-1.266788	0.886334
H	1.829378	-1.266583	-0.886434
O	1.923695	0.619273	0.000172
H	2.891827	0.589999	0.000215

****

234 (1pyrenemethanol,äÇ Tweezer, noH-bond)
scf done: -4770.425037

Au	21.047005	54.533311	54.331682
Au	24.885560	55.994943	59.870831
C	24.913844	58.037608	59.650084
C	24.749944	54.008835	59.970344
C	21.128001	56.478822	53.667300
C	21.079256	52.683506	55.066683
N	20.311316	57.501766	54.031869
N	25.594974	58.745249	58.711423
N	24.085273	58.922009	60.265989
N	22.082368	57.023306	52.867769
C	22.647090	59.403334	52.035578
N	22.891634	51.265791	58.039095
C	21.195856	51.573159	55.581970
C	19.094060	60.418848	54.319903
C	22.905082	61.795213	51.536877
C	25.304340	63.591566	57.423846
C	26.593138	58.108707	57.840296
C	22.488824	49.023604	58.031124
C	22.249121	61.471510	60.635983
C	23.829114	62.508824	59.086145
C	24.935001	62.436970	58.178877
C	19.411520	62.726947	53.762834
C	25.650509	61.218527	57.957061
C	21.872873	58.396354	52.715910
C	21.919223	47.845350	57.543333
C	18.680900	61.749177	54.443251
C	20.538852	62.409372	52.995537
C	21.478022	62.636816	60.698153
C	22.432020	63.147962	51.651996
C	21.923530	63.755849	59.987947
C	25.201811	60.084818	58.725984
C	28.008639	58.187005	58.409704
C	24.200838	60.185402	59.684782
C	19.174915	57.281484	54.934925
C	20.222882	60.042877	53.572458
C	23.416192	61.380001	59.859428
C	22.157534	60.742193	52.149326
C	21.300013	63.440063	52.343535
C	23.840239	59.171259	51.329616
C	23.369947	49.332546	59.143322
C	24.558805	64.809114	57.596616
C	23.976108	48.556819	60.138989
C	23.145173	56.202661	52.276248
C	20.971753	61.052319	52.890402
C	26.699808	61.203389	57.022889

C	24.797948	49.161664	61.096382
C	20.735086	58.700530	53.454644
C	23.296244	58.540656	61.449307
C	21.409405	50.323893	56.233651
C	24.605240	52.788265	60.011556
C	24.580995	60.200505	50.739202
C	17.479831	62.158316	55.314605
C	24.993563	50.558069	61.030536
C	23.587117	50.730695	59.104989
C	26.349249	63.514604	56.494924
C	20.855285	49.111072	55.780441
C	23.073707	63.716956	59.188897
C	22.225081	50.252875	57.377205
C	23.488275	64.866441	58.430925
C	28.169575	62.290222	55.212252
C	17.846718	57.130561	54.191911
C	27.056287	62.329441	56.273754
C	20.474102	46.559096	55.894789
C	21.090254	47.874678	56.411195
C	24.094135	61.505877	50.855084
C	25.494702	48.364594	62.215924
C	22.901612	55.888205	50.799505
C	25.899546	59.936985	49.990022
C	24.405622	51.377250	60.052695
C	20.150609	62.699369	61.473870
C	16.659691	56.967512	55.153192
C	20.203134	63.846145	62.507886
C	21.090568	57.663976	62.349510
C	25.181485	46.858974	62.142943
C	24.053993	55.076967	50.197662
C	21.921310	57.974353	61.101535
C	29.032819	57.571871	57.449559
C	30.462522	57.640727	57.990320
C	19.001587	62.961352	60.471408
C	16.745027	55.720819	56.042025
C	19.844079	61.388512	62.221398
C	23.842114	54.767638	48.714062
C	27.049596	60.664705	50.723219
C	25.787733	60.476579	48.545875
C	21.604936	45.575028	55.516176
C	19.588732	46.768070	54.652575
C	16.824440	60.954267	56.015266
C	16.414865	62.846174	54.431073
C	17.956624	63.145390	56.405976
C	26.247626	58.439045	49.916011
C	19.600648	45.929261	57.004036
C	25.022913	48.885837	63.592917
C	19.716521	57.087901	61.999115
C	27.026324	48.541560	62.100869
C	27.571012	62.673889	53.838705
C	29.276930	63.299626	55.590247
C	28.810086	60.896231	55.081692
H	18.787633	46.604883	57.291235
H	19.155291	44.989189	56.656673

H	20.182035	45.707419	57.904291
H	22.240328	45.995301	54.729084
H	22.246323	45.342112	56.371870
H	21.186772	44.630350	55.148046
H	20.155891	47.182021	53.812177
H	19.174329	45.807195	54.328374
H	18.746630	47.436922	54.859696
H	25.697043	46.333613	62.954149
H	24.109822	46.659958	62.251113
H	25.518823	46.418005	61.198756
H	25.260233	49.945029	63.731329
H	23.939185	48.770329	63.703385
H	25.509060	48.328149	64.402455
H	27.538870	47.980489	62.891555
H	27.389828	48.176349	61.134206
H	27.323732	49.590572	62.192889
H	22.858008	52.243294	57.791442
H	20.226899	49.163791	54.900330
H	22.125492	46.907132	58.049690
H	23.797026	47.488000	60.150548
H	25.626313	51.045567	61.764575
H	26.778793	61.977495	53.546537
H	27.143267	63.681012	53.845522
H	28.345886	62.646982	53.064379
H	29.583927	60.915789	54.307615
H	29.287514	60.576963	56.013965
H	28.076097	60.136041	54.792989
H	29.720624	63.048262	56.559582
H	30.074702	63.290937	54.838812
H	28.891647	64.321868	55.654176
H	30.770350	58.678781	58.165358
H	30.549907	57.104522	58.942309
H	31.176497	57.194529	57.289854
H	28.759618	56.526984	57.252935
H	28.980511	58.090817	56.482410
H	28.029540	57.657472	59.370183
H	28.269920	59.232753	58.618576
H	26.284978	57.071646	57.703267
H	26.519193	58.569865	56.857660
H	27.251044	60.292455	56.871555
H	26.594605	64.408476	55.928963
H	19.814613	56.154090	61.433724
H	19.130531	56.872012	62.898711
H	19.137307	57.789522	61.386323
H	20.964414	58.579137	62.944257
H	21.636360	56.953486	62.984327
H	21.384840	58.678586	60.457418
H	22.065442	57.064560	60.509321
H	23.228237	59.416382	62.097826
H	23.882016	57.790284	61.984931
H	21.907476	60.601563	61.168420
H	21.358656	64.682899	60.022267
H	22.916918	65.785130	58.535389
H	24.860150	65.681920	57.023210

H	18.041362	63.022372	60.996324
H	19.143518	63.898302	59.924185
H	18.933941	62.151560	59.736675
H	18.895412	61.482569	62.759487
H	19.748347	60.540024	61.535732
H	20.618306	61.150462	62.958553
H	20.364150	64.818909	62.033495
H	19.260116	63.901281	63.063412
H	21.012675	63.686840	63.228225
H	24.207871	58.165148	51.242186
H	24.641695	62.332532	50.412124
H	25.599408	61.554171	48.524677
H	26.718800	60.292391	47.998115
H	24.972557	59.983141	48.005856
H	27.166634	60.282894	51.742826
H	27.997752	60.512080	50.194980
H	26.873049	61.742478	50.787858
H	27.186593	58.304834	49.369512
H	26.384760	58.000799	50.910580
H	25.476138	57.867713	49.389434
H	24.674591	54.185307	48.305279
H	22.923181	54.190785	48.558844
H	23.757856	55.689055	48.125369
H	24.994378	55.630042	50.326367
H	24.167884	54.139955	50.758629
H	22.762421	56.817529	50.234102
H	21.963173	55.326189	50.717708
H	24.099758	56.704859	52.445227
H	23.169499	55.274389	52.850380
H	20.953314	64.466581	52.428643
H	23.007461	63.936071	51.173483
H	19.120827	63.771130	53.832629
H	18.530424	59.660628	54.832346
H	19.407852	56.373641	55.490040
H	19.155396	58.092950	55.662766
H	17.685123	58.000546	53.544535
H	17.918972	56.254556	53.535231
H	16.878353	54.814941	55.439344
H	17.583768	55.772379	56.744645
H	15.831656	55.598059	56.633842
H	16.578871	57.863705	55.783829
H	15.738307	56.927632	54.559753
H	16.424101	60.232594	55.295732
H	17.529656	60.433167	56.671767
H	15.988860	61.296826	56.634188
H	16.059644	62.168151	53.647640
H	15.552431	63.147349	55.036651
H	16.808164	63.743595	53.943674
H	18.708386	62.681090	57.051924
H	18.398017	64.049942	55.976674
H	17.114269	63.453321	57.035894
C	22.874044	62.262699	55.471800
C	23.763085	61.306247	54.989022
C	23.647619	59.961183	55.373370

C	22.597459	59.576518	56.259512
C	21.680430	60.563744	56.735836
C	21.844055	61.899156	56.336953
C	24.553758	58.948807	54.910047
C	22.461491	58.211478	56.662390
C	23.396579	57.227139	56.209381
C	24.442271	57.651247	55.314670
C	23.253353	55.884406	56.632404
C	22.189160	55.544749	57.475722
C	21.262743	56.490528	57.904987
C	21.381965	57.833516	57.518868
C	20.458139	58.843941	57.961330
C	20.608835	60.148714	57.599243
H	19.914682	60.902881	57.957597
H	19.637299	58.541789	58.606649
H	25.343614	59.242121	54.223947
H	22.982054	63.298343	55.168957
H	24.557044	61.595491	54.308555
H	21.154198	62.649759	56.708955
H	25.168911	56.919784	54.980757
H	22.078848	54.512434	57.791695
H	20.443615	56.191744	58.553158
C	24.218254	54.807385	56.180469
H	23.811672	53.822399	56.437186
H	24.319524	54.821444	55.090502
O	25.542583	54.964457	56.700400
H	25.501498	54.758143	57.649192

****

234 (1pyrenemethanol,äÇ Tweezer, withH-bond)
scf done: -4770.428339

Au	19.751310	65.102747	39.523682
Au	23.672687	69.980497	35.798100
C	25.023423	68.518534	35.308780
C	22.256305	71.200963	36.491406
C	20.430251	63.434684	38.533070
C	19.152451	66.820871	40.329491
N	20.269323	63.171874	37.209043
N	25.546201	67.632086	36.196722
N	25.401015	68.092911	34.076817
N	21.289525	62.493941	39.004286
C	22.678825	60.583097	37.952446
N	19.583692	70.524218	39.268160
C	18.753515	67.935513	40.659556
C	20.482256	61.741028	34.385516
C	23.819302	58.884099	36.591845
C	28.168172	63.421824	35.464927
C	25.307046	67.756290	37.642700
C	18.076926	71.646826	40.563608
C	26.351460	66.122529	31.779644
C	27.278066	64.831278	33.636474
C	27.486729	64.600712	35.034368
C	21.605083	60.025137	33.149225

C	26.986486	65.498606	36.027343
C	21.711998	61.651991	37.971928
C	17.105010	71.779198	41.563513
C	20.659950	61.053783	33.180082
C	22.341671	59.660006	34.284111
C	26.755595	65.158882	30.849937
C	24.019413	58.232771	35.325399
C	27.456266	64.045551	31.323114
C	26.257860	66.635668	35.528610
C	26.401819	68.540468	38.364265
C	26.128981	66.904796	34.170998
C	19.410202	64.006237	36.357818
C	21.188416	61.415538	35.555236
C	26.593676	65.992319	33.157141
C	22.864501	59.942626	36.685968
C	23.311868	58.600673	34.225657
C	23.453066	60.164407	39.048632
C	18.691261	72.588959	39.651759
C	28.638005	62.487342	34.478434
C	18.519739	73.962800	39.444607
C	21.671923	62.462256	40.422346
C	22.130434	60.340506	35.522070
C	27.212255	65.214277	37.383547
C	19.269951	74.624118	38.468082
C	21.037728	62.070884	36.830181
C	25.040586	68.849626	32.869614
C	18.248986	69.228064	40.985370
C	21.384695	71.794937	37.124935
C	24.387272	59.125303	38.960454
C	19.812991	61.363212	31.932234
C	20.196519	73.871397	37.716681
C	19.622032	71.850947	38.877497
C	28.335701	63.170986	36.833193
C	17.276955	69.410442	41.983031
C	27.725295	63.863256	32.686001
C	18.655931	70.385375	40.284840
C	28.421226	62.694830	33.153358
C	28.042461	63.722457	39.301518
C	18.007179	63.433939	36.162537
C	27.870362	64.056608	37.809610
C	15.639910	70.733728	43.411615
C	16.697652	70.659592	42.294165
C	24.549036	58.495772	37.723452
C	19.127789	76.131726	38.185816
C	22.957468	63.239899	40.708440
C	25.182330	58.636497	40.184895
C	20.405121	72.492807	37.890857
C	26.420176	65.276795	29.352686
C	17.176927	64.325000	35.231207
C	25.531869	64.078830	28.944058
C	23.395628	69.133419	30.960641
C	18.696757	76.344594	36.716386
C	23.364880	63.155963	42.182593
C	23.718733	68.387196	32.258287

C	26.137807	68.608158	39.872646
C	27.216043	69.390769	40.624913
C	25.654861	66.571234	29.020750
C	15.782154	63.757539	34.961044
C	27.724615	65.258797	28.524541
C	24.647136	63.937698	42.476687
C	25.042762	59.584292	41.391191
C	26.684015	58.519637	39.841167
C	15.112167	72.164703	43.621685
C	16.259833	70.251487	44.743277
C	20.724394	61.600384	30.708091
C	18.930722	62.612017	32.116707
C	18.893388	60.150798	31.655742
C	24.645819	57.243380	40.588901
C	14.439722	69.828406	43.050805
C	20.483624	76.834685	38.426486
C	22.077646	68.670569	30.335169
C	18.077148	76.798123	39.092742
C	27.598641	64.877439	40.218024
C	27.179266	62.480576	39.625455
C	29.524311	63.409196	39.606254
H	14.738923	68.782507	42.933325
H	13.676312	69.869354	43.837186
H	13.977593	70.149713	42.111001
H	14.627566	72.556555	42.720919
H	14.367534	72.171286	44.425118
H	15.912258	72.855613	43.908470
H	16.614854	69.218567	44.677106
H	17.112204	70.878624	45.026434
H	15.519889	70.297433	45.551511
H	17.734941	75.859336	36.517679
H	18.590224	77.413949	36.497518
H	19.427903	75.933787	36.013460
H	21.269442	76.439354	37.775680
H	20.399640	77.910379	38.229987
H	20.812701	76.702944	39.462956
H	18.339808	76.710795	40.152499
H	18.008265	77.865832	38.857782
H	17.081501	76.364610	38.949492
H	20.192916	69.781425	38.920979
H	16.961158	68.525137	42.524865
H	16.682282	72.758656	41.755502
H	17.798239	74.495624	40.053230
H	20.797047	74.364291	36.959436
H	26.535044	65.107943	40.101873
H	27.756873	64.601907	41.265274
H	28.172837	65.790038	40.026351
H	29.888360	62.545156	39.042544
H	30.163456	64.263906	39.360599
H	29.652581	63.184236	40.670971
H	26.115658	62.687898	39.465482
H	27.449908	61.631809	38.991091
H	27.314947	62.178272	40.669866
H	27.005635	69.426741	41.698993

H	28.203728	68.932612	40.492829
H	27.278318	70.423304	40.262458
H	25.156696	69.069562	40.045550
H	26.074899	67.588287	40.276782
H	26.438391	69.553036	37.943980
H	27.380923	68.082693	38.172862
H	24.344614	68.256716	37.760578
H	25.178824	66.753544	38.046822
H	26.867215	65.908837	38.127836
H	28.837959	62.253182	37.124415
H	21.864562	69.208910	29.405515
H	22.106470	67.599518	30.101194
H	21.239161	68.838961	31.019788
H	24.213303	68.988563	30.241323
H	23.348485	70.211911	31.162149
H	23.751508	67.307651	32.074322
H	22.925351	68.548669	32.996235
H	25.871603	68.778408	32.164950
H	24.965684	69.896521	33.171681
H	25.805211	66.977609	31.424078
H	27.793809	63.279544	30.631083
H	28.767320	61.973894	32.417340
H	29.160974	61.598017	34.820331
H	25.442278	66.609495	27.947515
H	24.695927	66.623269	29.546896
H	26.236264	67.464778	29.272105
H	28.293320	64.336200	28.674887
H	27.497893	65.337186	27.455187
H	28.372462	66.099009	28.796616
H	26.031703	63.121256	29.119184
H	24.596111	64.078506	29.513777
H	25.280891	64.134278	27.878584
H	23.331861	60.660491	39.995256
H	25.254971	57.677824	37.616343
H	26.870295	57.803587	39.035562
H	27.094438	59.486073	39.534144
H	27.244965	58.177033	40.717728
H	24.011928	59.635847	41.756738
H	25.659608	59.221637	42.219672
H	25.375439	60.598820	41.148485
H	23.582162	57.292108	40.845693
H	24.760308	56.519918	39.775228
H	25.189524	56.860314	41.460253
H	25.488613	63.546319	41.895432
H	24.530285	64.997291	42.221031
H	24.916731	63.879301	43.536497
H	22.546019	63.538863	42.805477
H	23.504263	62.103160	42.463792
H	22.789625	64.286299	40.426905
H	23.769035	62.863029	40.073417
H	20.842488	62.907329	40.975693
H	21.741944	61.419107	40.735664
H	23.466937	58.097809	33.274657
H	24.751145	57.431041	35.269961

H	21.778484	59.471616	32.230971
H	19.766674	62.542108	34.413168
H	19.346675	64.979427	36.844441
H	19.925064	64.169071	35.411313
H	18.066092	62.416647	35.756009
H	17.520302	63.354774	37.142216
H	15.216082	63.641211	35.892414
H	15.205527	64.413258	34.300046
H	15.841248	62.771567	34.484384
H	17.091350	65.326148	35.673956
H	17.712758	64.455728	34.281378
H	20.115867	61.789784	29.816606
H	21.370234	62.467073	30.870483
H	21.361752	60.737791	30.492029
H	18.374817	62.810459	31.194537
H	18.194121	62.478755	32.915582
H	19.530671	63.499859	32.341717
H	18.231907	59.958530	32.507439
H	18.268303	60.337457	30.774813
H	19.472022	59.240180	31.470046
C	25.028571	62.223510	35.118480
C	24.669468	62.946630	36.252319
C	23.774806	64.025723	36.161928
C	23.224754	64.370950	34.891278
C	23.613338	63.631118	33.731842
C	24.515612	62.565210	33.868732
C	23.383109	64.800294	37.303651
C	22.278819	65.437818	34.784301
C	21.888423	66.180253	35.946199
C	22.489925	65.826569	37.205924
C	20.910034	67.199801	35.823909
C	20.354000	67.459128	34.566201
C	20.730829	66.745917	33.430870
C	21.697440	65.733566	33.511544
C	22.130093	64.992903	32.356532
C	23.053503	63.997007	32.460795
H	23.377876	63.448424	31.581106
H	21.700348	65.248638	31.391546
H	23.812793	64.546642	38.267327
H	25.718771	61.391724	35.207387
H	25.087091	62.681904	37.218154
H	24.806855	62.002471	32.986922
H	22.222306	66.400564	38.083850
H	19.603103	68.239808	34.477741
H	20.283004	66.977921	32.468657
C	20.343786	67.933789	37.024058
H	19.600992	68.665661	36.679251
H	19.819991	67.226769	37.674697
O	21.304825	68.571872	37.870359
H	21.692468	69.315772	37.372348
